# The Influence of Polyploidy on the Evolution of Yeast Grown in a Sub-Optimal Carbon Source

**DOI:** 10.1093/molbev/msx205

**Published:** 2017-07-24

**Authors:** Amber L. Scott, Phillip A. Richmond, Robin D. Dowell, Anna M. Selmecki

**Affiliations:** 1 Department of Molecular, Cellular, and Developmental Biology, University of Colorado, Boulder, CO; 2 BioFrontiers Institute, University of Colorado, Boulder, CO; 3 Department of Medical Microbiology and Immunology, Creighton University Medical School, Omaha, NE

**Keywords:** experimental evolution, polyploidy, genomics, yeast, adaptive evolution, expression data

## Abstract

Polyploidization events have occurred during the evolution of many fungi, plant, and animal species and are thought to contribute to speciation and tumorigenesis, however little is known about how ploidy level contributes to adaptation at the molecular level. Here we integrate whole genome sequencing, RNA expression analysis, and relative fitness of ∼100 evolved clones at three ploidy levels. Independent haploid, diploid, and tetraploid populations were grown in a low carbon environment for 250 generations. We demonstrate that the key adaptive mutation in the evolved clones is predicted by a gene expression signature of just five genes. All of the adaptive mutations identified encompass a narrow set of genes, however the tetraploid clones gain a broader spectrum of adaptive mutations than haploid or diploid clones. While many of the adaptive mutations occur in genes that encode proteins with known roles in glucose sensing and transport, we discover mutations in genes with no canonical role in carbon utilization (*IPT1* and *MOT3*), as well as identify novel dominant mutations in glucose signal transducers thought to only accumulate recessive mutations in carbon limited environments (*MTH1* and *RGT1*). We conclude that polyploid cells explore more genotypic and phenotypic space than lower ploidy cells. Our study provides strong evidence for the beneficial role of polyploidization events that occur during the evolution of many species and during tumorigenesis.

## Introduction

Whole genome duplication events resulting in polyploidy (more than a diploid set of chromosomes) have occurred throughout the evolution of animals, plants, and fungi and are considered one of the driving forces of evolutionary change (Otto and Whitton 2000; Sémon and Wolfe 2007; Van de Peer etal. 2009). Recent examples demonstrate that polyploidy can arise in otherwise haploid or diploid organisms, and this polyploidization event correlates with increased invasiveness and pathogenesis (Pandit etal. 2011; Zaragoza and Nielsen 2013; Gerstein etal. 2015). Polyploidy is frequently observed in human cancers: nearly 37% of all cancers undergo a genome duplication event at some point during their progression (Zack etal. 2013). Additionally, polyploid cells can promote tumorigenesis in several cancer model systems, while isogenic diploid cells do not (Fujiwara etal. 2005). Polyploidy is also frequently identified in human fungal pathogens, including those that cause life-threatening infections like *Cryptococcus neoformans*, *Candida albicans*, and *Saccharomyces cerevisiae* (Muller and McCusker 2009; Harrison etal. 2014; Storchova 2014; Berman 2016; Zhu etal. 2016). Despite the importance of polyploidy in evolution and adaptation, little is known about how increasing ploidy levels affect adaptation to a stressful environment on a molecular level.

Ploidy level changes represent one of the most rapid means in which an organism can access large-scale genotypic and phenotypic variation (King etal. 2012; Soltis etal. 2014). Indeed, polyploidization events often immediately result in extensive karyotypic variability (Mayer and Aguilera 1990; Bennett and Johnson 2003; Gerstein etal. 2006; Storchová etal. 2006; Sémon and Wolfe 2007; Hufton and Panopoulou 2009) and polyploid cells may explore fundamentally different regions of phenotypic space because the genome redundancy enables a more diverse set of mutations upon which selection can act (Van de Peer etal. 2009). Important theoretical research has identified scenarios in which a given ploidy level may be beneficial to an organism (Stebbins 1940; Otto and Whitton 2000), however we lack experimental validation of many of these scenarios.

In theory, polyploidy may promote the rate of adaptation by doubling the target size for beneficial mutations (Adams and Hansche 1974; Otto 2007; Gerstein and Otto 2009). Increased numbers of chromosome sets may also buffer the effects of deleterious mutations (Korona 1999; Otto and Whitton 2000; Thompson etal. 2006). These effects are also impacted by the degree of dominance of beneficial mutations; although increased gene copy number amplifies the target size for mutation, phenotypic changes will be masked if the mutations are recessive (Orr and Otto 1994; Otto and Whitton 2000; Anderson etal. 2004). Finally, in smaller populations the rate of generating mutations is limiting, therefore the increased target size of polyploid cells, relative to haploids, is predicted to increase the rate of adaptation (Otto and Whitton 2000; Zeyl etal. 2003).

Some of these theories have been confirmed experimentally, yet studies have focused almost exclusively on haploid and diploid yeast (Paquin and Adams 1983; Anderson etal. 2004; Zeyl 2004; Gerstein and Otto 2009). For example, haploid cells evolve faster than diploid cells in large populations, but when the population size is reduced there is no advantage to haploidy (Zeyl etal. 2003). Similarly, haploid cells adapt faster in an environment where recessive mutations are favored, and diploid cells adapt faster in an environment that requires dominant mutations (Anderson etal. 2003, 2004). The increased mutational target size of diploid cells has been shown to be adaptive in strains defective in mis-match repair (diploid mutators) compared to diploid nonmutators, but there is no adaptive advantage to haploid mutators over haploid nonmutators (Thompson etal. 2006). Finally, the fitness effect of a given mutation is assumed to be equal across all ploidy levels, however recent experimental evidence suggests that this is not the case for all mutations (Gerstein 2012; Selmecki etal. 2015; Sellis etal. 2016). Ultimately, more examples are needed with cells of different ploidy levels and different growth environments in order to completely understand the degree to which the fitness landscape is altered by ploidy (Otto and Whitton 2000).

Witnessing spontaneous polyploidization events and following their evolutionary trajectories is difficult in nature, but it is possible in laboratory-controlled experiments with single-celled organisms. Polyploidization (diploid to polyploid) is observed in *C. albicans* during antifungal drug treatment (Harrison etal. 2014) and diploidization (haploid to diploid) is observed in *S. cerevisiae* during growth in rich medium and high salt (Gerstein etal. 2006, 2008), as well as after transformation and selection in low glucose medium (Venkataram etal. 2016). Additionally, the effect of ploidy on adaptation has been analyzed with many invitro evolution experiments that compare initially isogenic haploid and diploid strains (Anderson etal. 2003; Zeyl etal. 2003; Anderson etal. 2004; Gerstein etal. 2006; Thompson etal. 2006; Gresham etal. 2008; Gerstein etal. 2011; Wenger etal. 2011; Lang etal. 2013; Zörgö etal. 2013; Frenkel etal. 2014; Tamari etal. 2016), however these studies do not compare the adaptive genotype to phenotype at the whole genome and transcriptome level across haploid, diploid, and polyploid levels.

Our recent study has used invitro evolution with *S. cerevisiae* to test the effect of polyploidy on the rate and dynamics of adaptation (Selmecki etal. 2015). We passaged isogenic haploid (1N), diploid (2N), and tetraploid (4N) yeast strains in raffinose medium for 250 generations and isolated single colony clones for fitness and whole genome sequence analysis. All evolved clones had a significant increase in competitive fitness compared to their ancestor, indicating that they adapt to growth in raffinose medium, but the 4N-evolved clones undergo the largest change in fitness (Selmecki etal. 2015). The rapid adaptation of 4N-evolved clones is correlated with significant genome changes. We have quantified single nucleotide polymorphism (SNPs), small insertions/deletions (indels), gene copy number variations (CNVs), and ploidy level in 22 evolved haploid, 24 evolved diploid, and 28 evolved tetraploid clones using whole genome sequencing (WGS), comparative genome hybridization microarray (aCGH), and flow cytometry (Selmecki etal. 2015). 4N-evolved clones acquire significantly more CNVs, SNPs and indels than lower ploidy clones. Annotation of the genomic variants identifies distinct classes of mutations (synonymous, nonsynonymous, and intergenic) that occur at each ploidy (supplementary figure S1 & supplementary text S1, Supplementary Material online). Additionally, many of the 4N-evolved clones reduce ploidy to near-triploid or near-diploid, while the 1N-evolved and 2N-evolved clones never change ploidy or acquire aneuploidy (Selmecki etal. 2015). This work suggests distinct mechanisms of adaptation for each ploidy level.

Here we use transcriptome analysis to identify the mutations underlying adaptation to raffinose for all three ploidy levels. We focus on the gene expression patterns acquired during invitro evolution and find that only a small number of genes have robust differential expression in the evolved clones. The expression signature of just five key glucose-inducible genes enables us to determine the specific mutation responsible for adaptation to growth in raffinose medium in ∼100 evolved clones. Interestingly, gene expression patterns for all evolved clones cluster according to one key adaptive mutation, despite additional background mutations and underlying karyotype or ploidy level. We find that the combined polyploid lineages have a broader spectrum of these key adaptive mutations relative to isogenic diploid and haploid lineages. Our work supports that polyploid cells can rapidly adapt to a novel environment due to an increased sampling of these key adaptive mutations.

## Results

### Glucose Transport Is Upregulated in the Evolved Clones

**Fig. 1.**
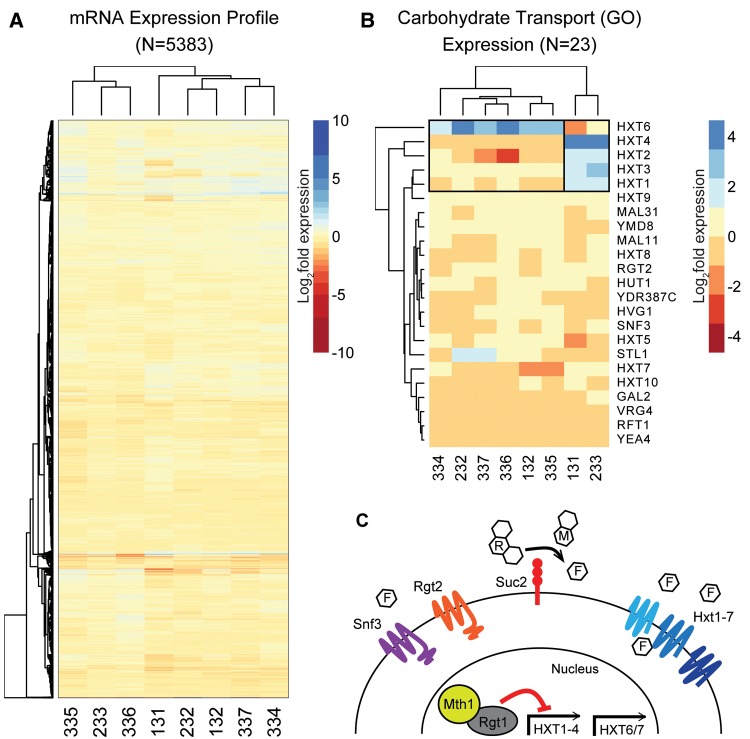
Carbohydrate transport drives the expression differences in the evolved clones. (*A*) Log_2_-transformed RNA-seq gene expression ratio between evolved clones (131, 132, 232, 233, 334, 335, 336, and 337) and the diploid ancestral strain. Hierarchical clustering was performed for both genes (*y*-axis, *n* = 5,383) and evolved clones (*x*-axis). (*B*) Log_2_-transformed RNA-seq gene expression of genes annotated with the GO-function of carbohydrate transport. The evolved clones cluster in two clades driven by overexpression of either the hexose transporters 1 through 4 (*HXT1-4*) or the *HXT6* transporter. (*C*) Graphical summary of glucose sensing and uptake in yeast (see supplementary text S2, Supplementary Material online for a brief review of glucose sensing pathway in yeast).

To understand the mechanisms by which strains of differing ploidy level adapted to growth in raffinose medium, we examined gene expression changes in the evolved clones after evolution. We performed a pilot RNA sequencing (RNA-seq) experiment in which we compared the gene expression in eight evolved clones (two 1N, two 2N, and four 4N) to the diploid ancestral strain (fig. 1*A*). Clones isolated from 1N-evolved, 2N-evolved, and 4N-evolved populations are denoted with strain identifiers in the 100 s, 200 s, and 300 s, respectively. We chose isolates for this pilot RNA-seq analysis based on their fitness relative to their ancestor (competitive fitness assays performed previously; Selmecki etal. 2015), and then for each ploidy level we selected one clone that did not carry the *HXT6/7* amplification (*HXT6/7*^amp^) (quantified previously; Selmecki etal. 2015). We selected one 4 N-evolved (337) clone that remained euploid and tetraploid; the other three 4N-evolved clones (334, 335, 336) became aneuploid and had ploidy ranging from near-triploid to greater than tetraploid (supplementary fig. S2, Supplementary Material online). In yeast, DNA levels result in proportional RNA levels (Torres etal. 2007). Our data confirmed that whole chromosome aneuploidies and segmental aneuploidies gave rise to systematically altered expression levels (clones 334, 335, 336), however the magnitude of observed fold changes were small. This is consistent with the fact that gains or losses of a chromosome in a tetraploid background result in a small change in DNA, proportional to base ploidy level. There were few major alterations in the gene expression in the evolved clones: on average only 26 genes were significantly differentially expressed in the evolved clones compared to the diploid ancestor in raffinose medium (fig. 1*A*).

We performed gene ontology (GO) term enrichment on the genes that were differentially expressed between the diploid ancestor and each evolved clone to determine the pathways that contributed to the adaptation. Two terms were commonly enriched in the evolved clones: phosphate ion transport genes were commonly downregulated and carbohydrate transport genes were frequently upregulated in the evolved clones relative to the diploid ancestor. Phosphate ion transport was decreased in all of the evolved clones, primarily driven by the overexpression of *PHO84* and *PHO89* in the diploid ancestor (supplementary fig. S3*A*, Supplementary Material online). We measured, by qRT-PCR, the expression of *PHO84* in the ancestral haploid and tetraploid strains. Only the diploid ancestor consistently had elevated *PHO84*, though with high variability (supplementary fig. S3*B*, Supplementary Material online). Regulation of the phosphate transport genes is controlled by a hysteretic switch, which may contribute to variability in activation of the phosphate transporters (Raser and Shea 2006).

Consistent with adaptation to a carbon stress, the differentially expressed genes showed enrichment for carbohydrate transport, specifically the hexose transporters (fig. 1*B*–*C* and supplementary text S2, Supplementary Material online). We performed hierarchical clustering of the RNA expression of the subset of genes annotated as “carbohydrate transport” in the evolved clones relative to the diploid ancestor (fig. 1*B*). Of the genes annotated as carbohydrate transport genes by GO, only the hexose transporters were differentially expressed*.* We validated the RNA-seq expression of the hexose transporters in evolved clones by qRT-PCR: the evolved clones 131 and 233 had robust expression of *HXT2, HXT3*, and *HXT4* and the evolved clones 132, 334, 335, 336, and 337 over-expressed *HXT6/7*, consistent with the RNA-seq data (supplementary fig. S4, Supplementary Material online). qRT-PCR did not replicate the overexpression of *HXT1*, consistent with the role of Hxt1 in glucose transport only during high levels of extracellular glucose (Ozcan and Johnston 1999).

To test if ploidy level itself had an effect on the expression of the hexose transporters in different carbon sources, we measured the expression of *HXT1*, *HXT2*, *HXT3*, *HXT4*, *HXT6/7*, and *SUC2* in the ancestral haploid, diploid, and tetraploid strains across four carbon environments (SC + 0.1% glucose, SC + 2% glucose, SC + 2% galactose, and SC + 2% raffinose) (supplementary fig. S5, Supplementary Material online). Ploidy level itself did not impact the expression level of the hexose transporters. However, the carbon source did influence the expression level of the different transporters. Notably, the ancestral strains over-expressed *HXT6/7*, *HXT2*, and *SUC2* when grown in 2% raffinose medium (supplementary fig. S5*D*, Supplementary Material online). Additionally, galactose, a nonfermentable carbon, did not induce expression of any of the glucose transporters in the ancestral strains (supplementary fig. S5*B*, Supplementary Material online), as expected (Ozcan and Johnston 1999). These results indicated that the initial ploidy level does not influence the hexose transporter expression levels, and suggested that comparing all evolved clones to the diploid ancestor was adequate for subsequent analysis.

DNA sequence data indicated the evolved clones overexpressing *HXT2*, *HXT3*, and *HXT4* had mutations in glucose sensing and signal transduction (*SNF3* and *MTH1*) while the cluster up-regulating *HXT6/7* all had amplifications of the *HXT6/7* region, with the exception of 4 N-evolved clone 334 (supplementary table S1, Supplementary Material online). These data suggest that there are two distinct pathways to up-regulate glucose transport in yeast. Moreover, the expression pattern of just a small number of genes, the glucose-inducible genes, is indicative of the pathway of adaptation in the evolved clones.

### Evolved Clones Form Gene Expression Clusters Predictive of Adaptive Mutation

**Fig. 2.**
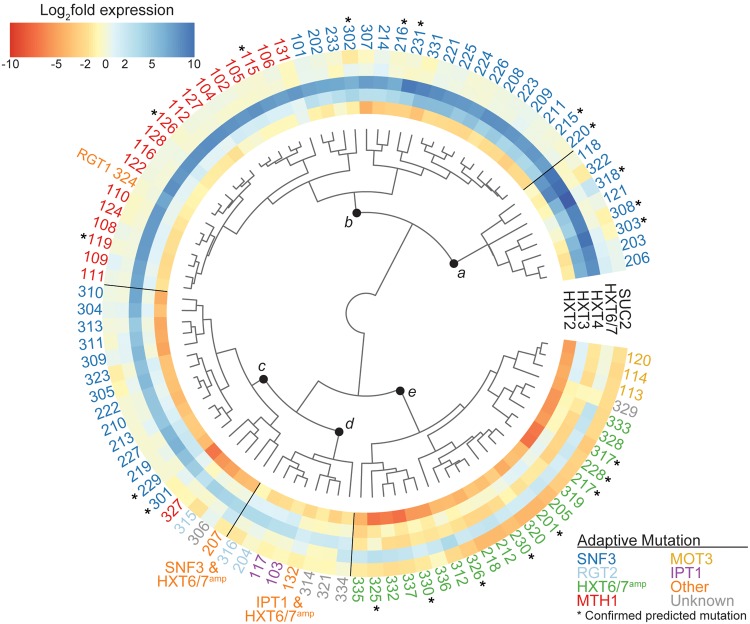
Expression pattern of glucose-inducible genes clusters the evolved clones by adaptive mutation. Heatmap and dendrogram of log_2_-transformed qRT-PCR gene expression ratio between haploid evolved clones (*n* = 27), diploid evolved clones (*n* = 32), and tetraploid evolved clones (*n* = 33) and the diploid ancestral strain. Evolved clones are clustered by the expression of a subset of genes involved in carbohydrate transport (*HXT2, HXT3, HXT4*, and *HXT6/7*) and metabolism (*SUC2*). The strain identifier color indicates the adaptive mutation determined for each evolved clone with whole genome or targeted Sanger sequencing: *SNF3* (blue), *RGT2* (light blue), *MTH1* (red), *HXT6/7^amp^* (green), *MOT3* (yellow), *IPT1* (purple), other (orange), and undetermined (grey). Asterisks (*) indicate the evolved clones for which targeted Sanger sequencing confirmed the mutation predicted by cluster analysis. Initial ploidy for each evolved clone is indicated by strain number: 1XX =1N, 2XX = 2N, and 3XX = 4N.

To determine whether a small panel of genes can identify the adaptive pathway in a larger number of evolved clones, we measured the gene expression pattern of the hexose transporters *HXT2, HXT3, HXT4, HXT6/7*, and the invertase *SUC2.* This set of genes was, by RNA-seq, diagnostic for the adaptive pathway in a set of evolved clones. Using qRT-PCR, we profiled the gene expression panel in 27 haploid evolved, 32 diploid evolved, and 37 tetraploid evolved clones that had high relative fitness in raffinose. This included clones previously genotyped using WGS and a subset of clones with no previous genotyping (supplementary table S1, Supplementary Material online). Hierarchical cluster analysis was performed on log_2_-transformed normalized gene expression values relative to the diploid ancestor grown in the same condition. The resultant gene panel dendrogram was plotted with a heatmap of the gene expression data in figure 2. While the RNA-seq data suggested two distinct patterns of *HXT* gene expression, the larger set of evolved clones clustered into five primary clades, labeled *a–e*. The optimal number of clades was selected by qualitative inspection of within-group variance estimation versus model complexity (supplementary fig. S6, Supplementary Material online).

To understand how the clusters of gene expression relate to the underlying mutations in the evolved clones, we determined the most likely primary adaptive mutation for each evolved clone for which we had WGS data. Adaptive mutations in genes not previously known to be involved in carbohydrate sensing and transport were determined based on multiple clones harboring unique mutations within the same gene. It should be noted that all mutations (SNPs and indels) that occurred in these genes in the 1N-evolved clones were homozygous, while all that occurred in the 2N-evolved and 4N-evolved clones were heterozygous, with allele frequencies in the 4N-evolved clones ranging from 25% to 50% (Selmecki etal. 2015). In five evolved clones, we were not able to determine a primary adaptive mutation from the WGS. The identity of adaptive mutations is indicated in figure 2 by the color of the clone identifier: *SNF3* (blue), *RGT2* (light blue), *HXT6/7^amp^* (green), *MTH1* (red), *MOT3* (yellow), *IPT1* (purple), other (orange) and unknown (grey). The clades identified by hierarchical cluster analysis were comprised primarily of evolved clones harboring adaptive mutations within the same gene, despite differences in additional background mutations and underlying karyotype.

To test whether our gene expression profiles were predictive of the underlying adaptive mutation, we examined the glucose-inducible gene expression profile in an additional 3 evolved haploid, 9 evolved diploid, and 9 evolved tetraploid clones for which WGS was not available. The adaptive mutation was predicted in each evolved clone based on how the clones clustered in the gene panel dendrogram (fig. 2, indicated by asterisks). Targeted Sanger sequencing of the candidate gene verified the predicted gene mutations and *HXT6/7* copy number was assessed for clones by either quantitative PCR or aCGH of the genomic DNA. In all 21 cases, the predicted mutation was confirmed (supplementary table S1, Supplementary Material online). Thus, this approach indicates that the adaptive mutation in evolved clones may be identified based on a particular phenotype, in this case gene expression.

### Activating Mutations in the Glucose Sensors: Snf3 and Rgt2

Two closely related glucose sensors, Snf3 and Rgt2, signal through downstream proteins to activate expression of the *HXT* genes in conditions of low and high extracellular glucose levels, respectively (fig. 1C) (Ozcan etal. 1996; Sabina and Johnston 2009). *SNF3* was the most commonly mutated gene across all three ploidy levels (found in 31 clones), while *RGT2* mutations were more rare, found only in 2N-evolved or 4N-evolved clones (3 clones total). Clones with mutations in these sensors clustered into multiple clades within the gene panel dendrogram (fig. 2). The clones with mutations in the gene encoding the glucose sensor Snf3 grouped within clades *a, b*, and *c* and varied in their levels of expression of *HXT2, HXT3*, and *HXT4*. The few clones with mutations in *RGT2* were primarily found in *clade d* and had only minimal over-expression of *HXT3* and *HXT4* when compared to the diploid ancestor*.* Moreover, evolved clones with *SNF3* mutations exhibited glucose independent activation of the hexose transporters (supplementary fig. S7, Supplementary Material online). These expression patterns were consistent with constitutive activation of glucose signal transduction through Snf3 or Rgt2.

**Fig. 3.**
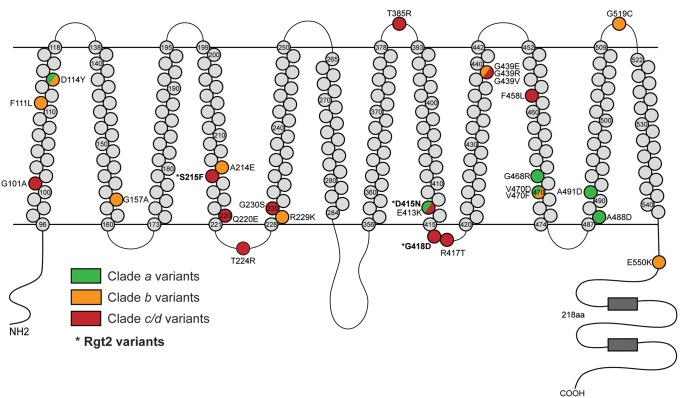
Mutations in genes encoding the glucose sensors, Snf3 and Rgt2, result in differing levels of glucose signal transduction. Diagram of Snf3 amino-acid substitutions that resulted from single nucleotide variations recovered in the evolved clones. Due to the high similarity between Snf3 and Rgt2 (Ozcan etal. 1996), Rgt2 variants, indicated with an asterisk (*), are also denoted in the diagram. The locations of transmembrane, cytosolic, and extracellular domains were predicted with TMHMM 2.0c (Krogh etal. 2001). Evolved clones with mutations in *SNF3* and *RGT2* clustered into multiple clades with varying levels in activation of the glucose-inducible genes: clade *a* (green, high activation), clade *b* (orange, moderate activation), and clade *c* or *d* (red, low activation).

The different clusters formed by *SNF3* mutants in the gene panel dendrogram were indicative of varying levels of downstream activation: high (clade *a*), moderate (clade *b*), and low (clade *c*). We diagramed the locations of the nonsynonymous mutations in Snf3 and colored the altered amino acids by the level of downstream activation (fig. 3: green, yellow, red; supplementary table S2, Supplementary Material online). In general, mutations with high activation were located near the cytoplasmic region of transmembrane domains 10 and 11 while mutations with low activation were located in the extracellular and cytoplasmic loops. The cluster analysis of many evolved clones suggests that unique gene expression patterns can result from different mutations in the same gene.

### *HXT6/7* Amplification Is More Common at Higher Ploidy Levels and Correlates with Increased Genome Stability in Tetraploids

Amplification of *HXT6/7* is a prominent mechanism of adaptation to growth in glucose limiting conditions (Brown etal. 1998; Dunham etal. 2002; Gresham etal. 2008; Kao and Sherlock 2008; Wenger etal. 2011) and we find that *HXT6/7^amp^* is more common with increasing ploidy (Selmecki etal. 2015). The majority of clones harboring *HXT6/7^amp^* clustered together in clade *e*, overexpressing *HXT6/7* while down regulating the other hexose transporters and *SUC2* compared to the diploid ancestor (fig. 2). We compared the ploidy level of the 4 N-evolved clones with *HXT6/7^amp^* to 4 N-evolved clones with other adaptive mutations and found that the *HXT6/7^amp^* clones had significantly higher ploidy levels than clones with *SNF3* mutations or other adaptive mutations (F(2,25) = 12.67, *P* = 1.6 × 10^−4^, ANOVA with post-hoc Tukey test) (supplementary fig. S8*A*, Supplementary Material online). Additionally, we found that the 2N-evolved clones with *HXT6/7^amp^* acquired fewer mutations per clone compared to 2N-evolved clones with adaptive mutations in other genes (*P* = 1.55 × 10^−3^, Welch two sample *t*-test, df = 13.47) (supplementary fig. S8*B*, Supplementary Material online). We did not observe a significant difference in the total number of mutations per clone in the 4N-evolved clones with *HXT6/7^amp^*compared to clones with mutations in other genes (supplementary fig. S8*B*, Supplementary Material online). Since the 4N-evolved clones with *HXT6/7^amp^*had a greater genome size, we corrected the number of mutations per clone by the base ploidy level. While there were fewer mutations per base ploidy level in the 4N-evolved clones with *HXT6/7^amp^*, the difference was not statistically significant (data not shown). Overall, these data suggest that adaptation through *HXT6/7^amp^* may decrease ploidy drive and increase genome stability in the 2N- and 4N-evolved clones.

### Novel Dominant Mutations in the Signal Transducers, Mth1 and Rgt1

**Fig. 4.**
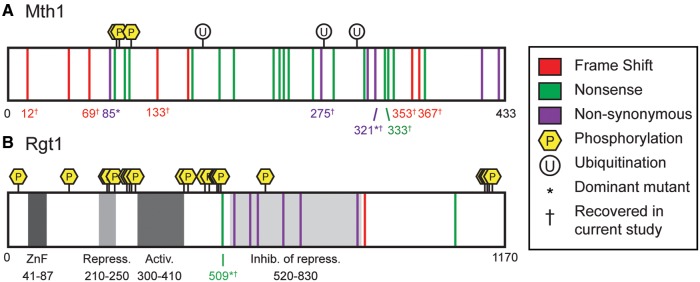
Mutations in *MTH1* and *RGT1* recovered from experimental evolution studies. Diagram of mutations identified in this study and previous publications in (*A*) *MTH1* and (*B*) *RGT1*. The type of mutation is indicated by color: frameshift (red), nonsense (green), and nonsynonymous (purple). Known domains in Rgt1 are indicated in grey (Polish etal. 2005). Known ubiquitiation (white, circle) and phosphorylation (yellow, hexagon) sites are also indicated (Cherry etal. 2012). Numbers indicate amino acid residues. For detailed information about the strains or populations with the indicated mutations see the supplementary tables S3 and S4, Supplementary Material online.

Several of the evolved clones had mutations in the signal transducer *MTH1*. With one exception, these clones all clustered together as a sub-clade within clade *b*, with moderate/high expression of *HXT3* and *HXT4*, consistent with Mth1 loss-of-function (fig. 2). The major outlier was the 4N-evolved clone 327 (ploidy is near-triploid; Selmecki etal. 2015), which harbored a heterozygous nonsynonymous mutation in *MTH1* and clustered in clade *c* (fig. 4*A* asterisk, C321F, supplementary table S3, Supplementary Material online). This clone had a different expression profile than the other Mth1 loss-of-function mutant clones, suggesting that this genotype did not fully abolish all Mth1 function. While dominant nondegradable *MTH1* mutants that result in constitutive repression of the hexose transporters were previously described (Schulte etal. 2000), this mutation is a dominant activating mutation in *MTH1*, causing constitutive over-expression of the hexose transporters (supplementary fig. S9*A*, Supplementary Material online).

We obtained a single evolved clone with a mutation in the gene encoding the negative regulator of glucose-inducible gene expression, Rgt1*.* This clone, 4N-evolved clone 324 (ploidy near-diploid, Selmecki etal. 2015), clustered with the *MTH1* mutants in clade *b* (fig. 2), consistent with loss of Rgt1 mediated repression of the glucose-inducible genes. Yet this mutation occurred in only 1 of 2 alleles, with a remaining wild type copy (*rgt1^S509stop^/RGT1*). The loss of a single *RGT1* allele does not result in haploinsufficiency (Marshall-Carlson etal. 1991; Dietzel etal. 2012), yet the heterozygous mutation that we recovered in *RGT1* leads to the dominant activation of the hexose transporters *HXT3* and *HXT4* in raffinose. The mutation resulted in a heterozygous truncation of the Rgt1 domain described as an inhibitor of repression (Polish etal. 2005) (fig. 4*B* asterisk, S509Stop, supplementary table S4, Supplementary Material online). This domain is thought to function as a self-inhibiter of the DNA binding domain of Rgt1. Thus, we expected that loss of this region would result in constitutive binding of Rgt1 and, as a result, constitutive repression. However, we saw the opposite effect: the expression profile of *rgt1*^S509stop^/*RGT1* phenocopied loss of *MTH1*.

Previous experimental evolution studies on haploid yeast populations in glucose-limiting conditions recovered mutations in *RGT1* that were presumed to be loss-of-function (Koschwanez etal. 2013; Kvitek and Sherlock 2013). The reported mutations within *RGT1* were located in the same inhibitor of repression domain described above (fig. 4*B*). However, since we did not recover haploid loss-of-function mutations in *RGT1*, we hypothesized that loss of *RGT1* would be detrimental to growth in raffinose. To test this, we deleted *RGT1* in the haploid ancestral strain. Multiple *rgt1*Δ strains displayed a growth defect on raffinose compared to the 4N-evolved clone 324 (supplementary fig. S9*C*, Supplementary Material online). Furthermore, we did not observe a difference in growth for *rgt1*Δ strains compared to the haploid ancestor in raffinose or glucose (supplementary fig. S9*B* and *C*, Supplementary Material online). These results suggest that the *rgt1^S509stop^*/*RGT1* mutation in 4N-evolved clone 324, as well as mutations in *RGT1* described in other studies, do not fully abolish Rgt1 function. Instead, these mutations may affect association with co-regulators, such as Mth1 or Ssn6/Tup1.

### Novel Mutations in *IPT1* and *MOT3* Improve Fitness in Raffinose Medium

The 1N-evolved clones 103, 117, and 132 each gained unique nonsynonymous or nonsense mutations in *IPT1*. Ipt1, an inositolphosphotransferase, is the final step in the biosynthesis of complex sphingolipids, the loss of which has been shown to improve antifungal drug resistance (Dickson etal. 1997; Hallstrom etal. 2001). It is improbable that the multiple mutations would occur in *IPT1* by chance (*P* = 6.2 × 10^−6^, exact binomial test), suggesting that mutations in *IPT1* are adaptive and under positive selection. The *IPT1* mutant clones slightly up-regulated *HXT3* and *HXT4* and clustered together in clade *d* (fig. 2). In a recent study that examined the fitness of gene deletions in several environments, loss of *IPT1* was predicted to have fitness benefits in low-glucose and low-sulfate conditions (Payen etal. 2016). Our results indicated that beneficial mutations in *IPT1* can arise during experimental evolution, and the unique nonsynonymous variants may provide mechanistic insight into its function.

Similarly, three 1N-evolved clones harbored mutations in *MOT3*, a transcription factor that has been shown to regulate a wide range of genes, including ergosterol biosynthetic genes and glucose transporters (Grishin etal. 1998; Hongay etal. 2002). As with *IPT1*, multiple hits in *MOT3* were unlikely to occur by chance (*P* = 5.0 × 10^−6^, exact bionomial test). Previously, overexpression of *MOT3* was shown to increase the transcription of *HXT2, HXT3, HXT4*, and *SUC2* (Grishin etal. 1998). The 1N-evolved clones 113, 114, and 120 had distinct frameshift (P268fs) and nonsense (R357Stop, K394Stop) mutations in *MOT3* (supplementary table S1, Supplementary Material online). These clones clustered together in clade *e*, adjacent to clones with *HXT6/7^amp^* and were characterized by up-regulation of *HXT3* and *HXT6/7* and down regulation of *HXT2, HXT4*, and *SUC2* (fig. 2).

In carbon limited environments, Mot3 forms a prion protein that represses Mot3 activity and the prion [*MOT3^+^*] functions to prime yeast cells for growth in low-carbon environments by switching from fermentative to oxidative metabolism and producing a *FLO11-*induced multicellular phenotype in some lineages (Halfmann etal. 2012; Holmes etal. 2013). The evolved clones with mutations in *MOT3* did not show evidence of prion formation by growth on glycerol glucosamine medium or multicellularity phenotypes (data not shown). Thus, it was unlikely that the *MOT3* mutants were adapting through prion formation. Despite the unknown role of Ipt1 and Mot3 in adaptation to growth in raffinose, these clones exhibited high levels of competitive fitness compared to the diploid ancestor (supplementary fig. S10*A*, Supplementary Material online).

### Adaptive Mutation Is a Better Separator of Evolved Clones Than Ploidy

**Fig. 5.**
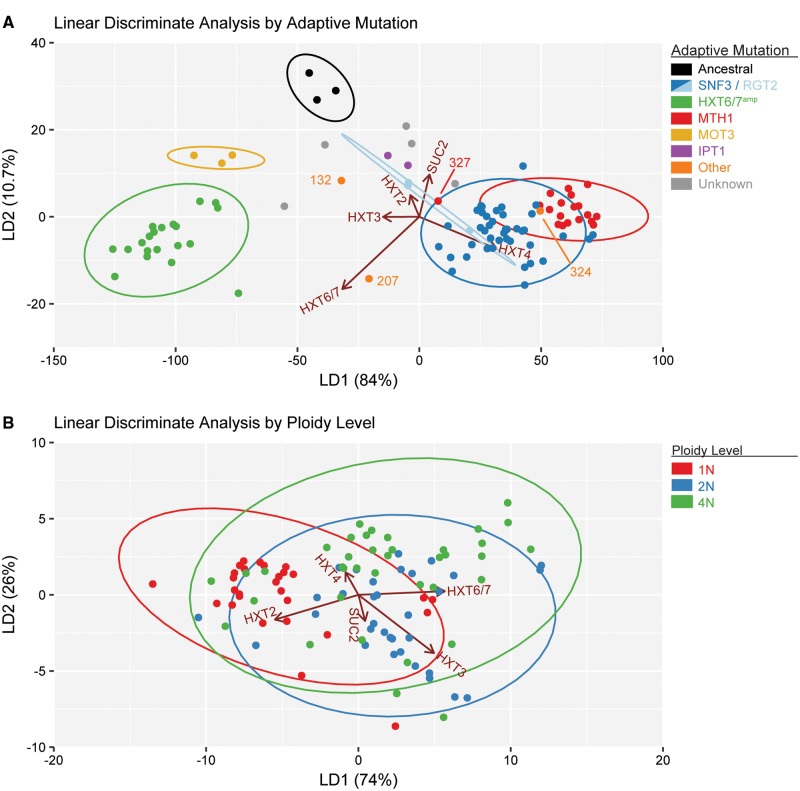
Linear discriminant analysis separates evolved clones into distinct populations by adaptive mutation. Linear discriminant analysis (LDA) of the log_2_-transformed gene expression ratio of the evolved clones relative to the diploid ancestral strain. Gene expression of *HXT2, HXT3, HXT4, HXT6/7*, and *SUC2* was measured by qRT-PCR and normalized to *ACT1* expression. (*A*) LDA of the evolved clones classified by adaptive mutation. The marker color indicates the major classes of adaptive mutations: parental (black), *SNF3* (blue), *RGT2* (light blue), *MTH1* (red), *HXT6/7^amp^* (green), *MOT3* (yellow), *IPT1* (purple), other (orange), and undetermined (grey). The ellipses denote the confidence interval of 95% for each major class of primary adaptive mutation. (*B*) LDA of the evolved clones classified by initial ploidy. The marker color indicates the initial ploidy: 1N (red), 2N (blue), and 4N (green). The ellipses denote the confidence interval of 95% for each ploidy level.

To examine the effect of underlying ploidy level on the gene expression profiles in the evolved clones, we performed linear discriminant analysis (LDA) on the glucose-inducible gene expression panel. LDA is a form of dimensionality reduction that is similar to principal component analysis (PCA), which models the data to emphasize the components that explain the most variation in the data set. However, unlike PCA, LDA attempts to model the difference between the data classes as well. We classified the clones by either primary adaptive mutation or initial ploidy (fig. 5). We found that LDA performed considerably better when the data were classified by adaptive mutation rather than initial ploidy. When the clones were classified by adaptive mutation the clones formed discrete groups (fig. 5*A*). When the evolved clones were classified instead by initial ploidy, the resulting groups were not well defined (fig. 5*B*). We did observe sub-clusters in the LDA within each ploidy level: for example, 1N-evolved clones with *MTH1* mutations clustered together, however the expression patterns in these clones were not predictive of the underling ploidy given the overlap of the 95% confidence intervals (fig. 5*B*, ellipses). These results confirm that the primary adaptive mutation is the main driver for the expression pattern of glucose-inducible genes in the evolved clones, while the initial ploidy influences the relative frequency of the different adaptive mutations acquired.

## Discussion

We integrated whole genome sequencing with gene expression data in order to understand the molecular mechanisms of adaptation across three different ploidy levels. We previously reported that 4N cells adapt more rapidly to raffinose medium than genetically identical 1N or 2N cells (Selmecki etal. 2015). While all evolved clones had significantly higher fitness than their progenitor in raffinose medium, it was unclear what mutation(s) were driving this adaptation. Whole genome sequence analysis identified significantly more SNPs, indels, CNVs, and ploidy changes in the 4N-evolved clones than in the 1N- or 2N-evolved clones (Selmecki etal. 2015), which made identification and validation of adaptive mutations in the 4N-evolved clones more challenging. In our RNA-seq analysis, we found that only a small number of genes showed robust differential expression in the evolved clones of all three ploidy levels, relative to the diploid ancestor. The expression signature of just five key glucose-inducible genes enabled us to determine the specific mutation responsible for adaptation to growth in raffinose medium in ∼100 evolved clones. Importantly, we accurately predicted the adaptive or driving mutation in clones with no available WGS data using only their gene expression phenotype. This phenotype-to-genotype approach could have broad implications for the identification of novel mutations that cause disease and cancer, including mutations in highly polyploid and aneuploid cancer genomes (Zack etal. 2013).

### Beneficial Mutations Identified by Transcriptional Phenotype

In our study, the primary mode of adaptation to raffinose medium at all ploidy levels was to increase glucose uptake by over-expression of the hexose transporters via adaptive mutations in the carbohydrate sensing and transport genes, similar to adaptation to low-glucose environments (Brown etal. 1998; Gresham etal. 2008; Kao and Sherlock 2008; Koschwanez etal. 2013; Kvitek and Sherlock 2013). We found two main mechanisms of adaptation: up-regulation of *HXT2*, *HXT3*, and *HXT4* primarily through mutations in *SNF3* and *MTH1* (fig. 2: clades *a–c*) or by up-regulation of *HXT6*/*7* primarily through mutations resulting in amplification of these two genes (fig. 2: clade *e*). This pattern of mutually exclusive transporter usage was observed in clones that adapted to growth in low glucose (Kvitek and Sherlock 2011). Importantly, these mutually exclusive transport mechanisms were utilized by clones from all three ploidy lineages, however the identity of adaptive mutations that they acquired varies by ploidy level (discussed below).

The gene expression phenotypes of key glucose-inducible genes and the analysis of ∼100 evolved clones enabled us to identify novel adaptive mutations that impact carbohydrate metabolism in yeast. Two genes, *IPT1* and *MOT3*, had no defined role in carbon utilization, and yet mutations in these genes resulted in the highest relative fitness of all 1N-evolved clones (supplementary fig. S10*A*, Supplementary Material online). One commonality between Ipt1 and Mot3 is they both function in the biosynthesis of plasma membrane constituents, specifically sphingolipids and ergosterol, respectively (Dickson etal. 1997; Hongay etal. 2002). This suggests that one mode of adaptation to nutrient limitation involves altering the plasma membrane or the endocytic pathway, including improved membrane organization, trafficking, recycling, and/or retention of the hexose transporters at the cell surface. In support of this, adaptive mutations were recently identified in genes encoding components of the endocytic pathway in yeast that had adapted to nitrogen limitation (Hong and Gresham 2014). Additionally, the use of tetraploid lineages enabled us to discover novel dominant mutations in the genes encoding the glucose signal transducers, Rgt1 and Mth1. We hypothesize that these mutations affect signaling by altering associations with co-regulators, such as Ssn6/Tup1. Theoretically, dominant mutations could be recovered in diploid lineages, however we only observed these rare dominant mutations in the tetraploid lineages, reflecting the broad spectrum of mutations observed within the 4N-evolved clones.

We also identified a large number of distinct mutations within the gene that encodes the glucose sensor Snf3. These mutations resulted in varying levels of activation of the glucose-inducible genes, highlighting specific protein domains of Snf3 which resulted in low, medium, or high glucose signal transduction (fig. 3). The ability of our gene expression panel to detect “fine tuning” of transcriptional pathways due to different mutations in the same gene will likely lead to a better understanding of the molecular function of this glucose sensor. Additionally, this suggests that natural selection has restricted the signaling capabilities of Snf3 resulting in lower levels of glucose signal transduction than this protein is capable of producing.

### Tetraploids Acquire a Broader Spectrum of Adaptive Mutations

**Fig. 6.**
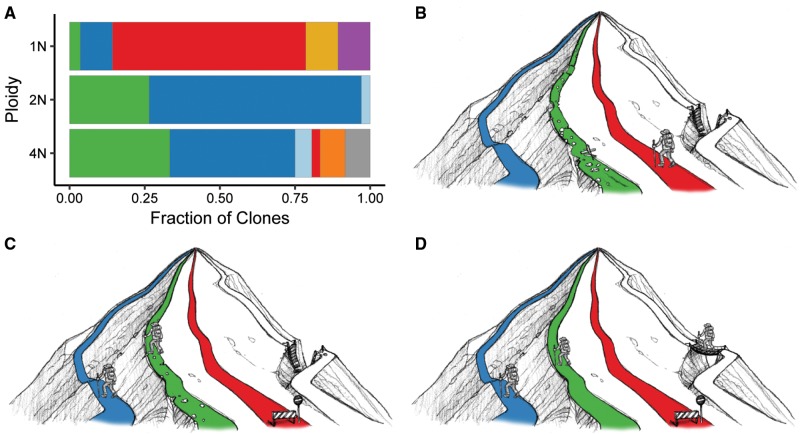
Pathways of adaptation to growth in raffinose medium for haploid, diploid, and tetraploid yeast. (*A*) The fraction of evolved clones derived from 1N, 2N, or 4N populations with the indicated adaptive mutation. The color denotes the adaptive mutation determined for each evolved clone with whole genome or targeted Sanger sequencing: *SNF3* (blue), *RGT1* (light blue), *MTH1* (red), *HXT6/7^amp^* (green), *MOT3* (yellow), *IPT1* (purple), other (orange), and undetermined (grey). Cartoon depicting the different adaptive pathways utilized by the (*B*) haploid, (*C*) diploid, and (*D*) tetraploid evolved clones during long-term growth in raffinose medium. Aneuploidy, only observed in the 4N-evolved populations, is represented by the white pathway. The hiker signifies the pathways most commonly utilized by clones at each starting ploidy level. Only most frequent mutation classes shown.

The identity of mutations acquired by strains of each ploidy level likely resulted from a combination of factors, including DNA copy number and physiological differences. Here, we found that the underlying ploidy level had a major effect on the spectrum of adaptive mutations acquired during growth in raffinose (fig. 6*A*). Not all pathways of adaptation were available to clones from different ploidy backgrounds. For example, the most common pathway of adaptation in the 1N-evolved clones was through loss-of-function mutations in the gene encoding the signal transducer Mth1, which was not observed in either the 2N- or 4N-evolved clones (fig. 6*B*). Conversely, the 2N-evolved and 4N-evolved clones adapted predominantly through mutations in the gene encoding the glucose sensor Snf3 or amplification of the genes encoding the glucose transporters Hxt6 and Hxt7 (fig. 6*C* and *D*). Some adaptive routes were open to all ploidy levels: we found nonsynonymous mutations in the genes encoding glucose sensors in 1N-evolved, 2N-evolved, and 4N-evolved clones. However, the beneficial effect of a mutation can differ depending on the ploidy of a cell (Gerstein 2012), and we previously showed that chromosome XIII aneuploidy provided a tetraploid-specific fitness benefit in raffinose medium (Selmecki etal. 2015). Ultimately, the 4N-evolved clones together acquired more mutations per clone (SNPs and indels) and a broader spectrum of adaptive mutations than the 1N- and 2N-evolved clones. This suggests that 4N-evolved clones explore more genotypic space and may explain why they adapt faster to raffinose medium than 1N- and 2N-evolved clones.

Amplifications of the *HXT6/7* region occur more often with increasing ploidy level, consistent with increasing amounts of homologous recombination in diploids and tetraploids (Storchová etal. 2006). *HXT6/7^amp^* arises as a result of ectopic gene conversion, which can result in extra chromosomal circular DNA, or unequal crossover, both of which occur during homologous recombination (Brown etal. 1998; Møller etal. 2015). Interestingly, we rarely detected point mutations in any of the *HXT* genes, indicating that modifying the copy number of the genes encoding the Hxt6 and Hxt7 transporters was favored over altering the function of a specific transporter. This is consistent with studies that show *HXT6/7* copy number can change rapidly (Møller etal. 2015; Mishra and Whetstine 2016). The ability to rapidly and reversibly respond to changing levels of glucose in the environment through *HXT6/7^amp^* represents an advantage to clones that gained *HXT6/7^amp^* compared to clones with other mutations that have similar fitness (supplementary fig. S10*B* and *C*, Supplementary Material online)*.* Thus, *HXT6/7^amp^* and its inherent reversibility may lead to better long-term survival in changing glucose conditions.

A significant physiological difference between 1N, 2N, and 4N yeast is the surface area to volume ratio (Storchová etal. 2006). Since higher ploidy cells have a lower surface area to volume ratio, they exhibit differential expression of cell surface components relative to haploids (de Godoy etal. 2008; Wu etal. 2010). A decreased surface area to volume ratio at higher ploidy levels and greater metabolic need may contribute to increased selection for the over-expression of nutrient transporters at higher ploidy. Thus, a fundamental difference in cell size is one force that may drive the differential spectrum of adaptive mutations with increasing ploidy. Tetraploid strains frequently undergo ploidy reduction by chromosome loss (Mayer and Aguilera 1990; Gerstein etal. 2006; Storchová etal. 2006; Gerstein etal. 2008; Selmecki etal. 2015; Voordeckers etal. 2015), suggesting that they are at least initially under selective pressure to reduce ploidy and cell size in many different environments. Interestingly, 4N-evolved clones with *HXT6/7^amp^* appear to have increased genome stability suggesting that *HXT6/7^amp^* may reduce the selective pressure on 4N cells for ploidy reduction (supplementary fig. S8*A*, Supplementary Material online).

Here we combined WGS data with the gene expression phenotype of key glucose-inducible genes to identify the beneficial mutations driving adaptation to growth in raffinose medium. Our previous mathematical modeling indicated that tetraploid lineages can accelerate the rate of adaptation by both an increased rate of beneficial mutations and increased strength of these mutations (Selmecki etal. 2015). However, here we found that evolved clones of all ploidy levels acquired mutations that alter the expression of key glucose-inducible genes in a well-defined way. Moreover, the specific adaptive mutation was a better predictor of glucose-inducible gene expression than karyotypic information. While ploidy did not alter the overall expression pattern of these key glucose-inducible genes, it did have a large impact the acquisition of adaptive mutations (fig. 6). Tetraploid yeast acquired a greater number of mutations and gained more diverse adaptive mutations than haploids and diploids, indicative of greater flexibility during adaptation. Even if polyploidy and/or aneuploidy is transient during adaptation (Rancati etal. 2008; Yona etal. 2012), the beneficial mutations acquired in the polyploid state can be preserved during ploidy reduction if they remain beneficial at lower ploidy levels. Our study provides strong evidence for the beneficial role of polyploidization events that occur during the somatic evolution of human pathogens and during tumorigenesis.

## Materials and Methods

### Strains Used in This Study

Ancestral strains and the evolved clones utilized in this study are listed in the supplementary table S1, Supplementary Material online. All strains used in this study were derived from the *Saccharomyces cerevisiae* S288c background. A detailed description of the ancestral strains and evolved clones can be found in (Selmecki etal. 2015). Briefly, isogenic haploid (1N), diploid (2N), and tetraploid (4N) ancestral strains were constructed and passaged for 250 generations in SC + 2% raffinose medium. A single colony clone was isolated from each population at the end of the invitro evolution experiment, by streaking populations out on agar plates containing SC + 2% raffinose. WGS, aCGH, flow cytometry, and relative fitness assays were performed on 22 haploid, 24 diploid, and 28 tetraploid clones (Selmecki etal. 2015). In this work, additional clones were selected for WGS and RNA sequencing analysis (131 and 132) and expression analysis (115, 119, 126, 201, 215, 216, 217, 220, 228, 229, 230, and 231). The *rgt1Δ* strain was produced replacing the entire *RGT1* ORF in the PY5999 (1N) ancestral strain with the pCORE cassette (Storici etal. 2001). Transformants carrying the pCORE cassette were selected on YPD + 200 µg/mL G418 (GoldBio), and four clones were selected for confirmation by PCR. A complete list of primers can be found in the Supplementary Materials online.

### Growth Conditions

For a detailed description of growth conditions used in the experimental evolution experiments, refer Selmecki etal. (2015). For the RNA expression analyses, yeast were grown to saturation at 30 °C at 300 rpm for 20 h in synthetic complete medium (SC)+2% raffinose. Cell density was determined by optical density (OD) and cells diluted to 0.2 ODU in SC + 2% raffinose. Cultures were grown at 30 °C at 300 rpm until mid-log growth (0.8–1.0 ODU). For glucose independent gene expression, cultures were grown to saturation in SC + 2% raffinose medium, and then diluted to 0.2 OD in SC + 2% galactose and harvested after growth for 6 h at 30 °C. RNA-seq cultures were grown in 100 ml of medium in a flask while qRT-PCR cultures were grown in 5 ml culture tubes. Cells were pelleted at 4 °C for 5 min at 5000 × *g*, washed with DEPC treated water, snap frozen and stored at −80 °C.

### Whole Genome Sequencing

Two 1N-evolved clones were sequenced in this study (131 and 132). DNA was isolated with phenol chloroform as described previously (Selmecki etal. 2015). Libraries were prepared using the NexteraXT DNA Sample Preparation Kit following the manufacturer’s instructions (Illumina). Libraries were sequenced using an Illumina MiSeq (Creighton University), and sequence alignment was performed using the S288c R63.1.1 reference genome as previously described (Selmecki etal. 2015). Variant calling was performed using SNPeff (v4.1 with default settings) (Cingolani etal. 2012). All variants previously called in Selmecki etal. (2015) were confirmed using SNPeff.

### RNA Isolation

RNA was isolated using the hot acid phenol RNA extraction method and RNA was DNase treated with RQ1 DNase at 37°C for 60 min (Promega, M6101). The DNase-treated RNA was extracted with phenol:chloroform:isoamyl alcohol 25:24:1 (Sigma, P2069) followed by chloroform extraction. The DNase-treated RNA was precipitated and the pellet was resuspended in 50 µl DEPC treated H_2_O. RNA quality was assayed by electrophoresis in MOPs gel or BioAnalyzer (BioRad). Total RNA was used for input into the RNA-seq library and RT-qPCR.

### RNA Library Construction

Poly(A) tailed RNA was isolated from total RNA with the Oligotex mRNA mini kit (Qiagen, 70022) following manufacturer’s directions. Strand specific RNA libraries were prepared from 100 ng Poly(A)^+^-RNA using the Illumina RNA ligation library protocol (Levin etal. 2010). The cDNA libraries were amplified with Phusion High-Fidelity DNA polymerase (NEB) and indexing primers compatible with Illumina sequencing. After amplification, libraries were size selected on a nondenaturing polyacrylamide gel for 250–400 bp fragments. Resultant libraries were assessed by BioAnalyzer before sequencing.

### RNA Sequencing Analysis

RNA-seq libraries were sequenced on 1 × 50 flow cell on an Illumina HiSeq2000 (University of Colorado). Libraries were sequenced to an average depth of 200 M reads per strain. Adaptor sequences and low quality reads were trimmed with trimmomatic (v0.32 ILLUMINACLIP:TruSeq2-SE.fa:2:30:10 LEADING:3 TRAILING:3 SLIDINGWINDOW:4:15 MINLEN:36) (Bolger etal. 2014). In order to remove ribosomal reads we first mapped the reads to a custom fasta file containing only the ribosomal gene region (chrXII:450486-459797) with Bowtie2 (v2.02, –sensitive-local) and all unmapped (nonribosomal reads) were output to an unmapped fastq file (Langmead and Salzberg 2012). The resulting nonribosomal reads were mapped to *S. cerevisiae* genome (R63.1.1, 2010-01-05) with Bowtie2 (v2.02, –sensitive-local) with the best mapping locations reported for each read (Engel etal. 2014). An average of 58 M reads mapped (a 95% mapping rate) per strain. SAM files were converted to BAM with samtools v0.1.19 for downstream use (Li etal. 2009). Reads that mapped unambiguously to transcripts were counted using HTSeq (v0.5.4p5, htseq-count -f bam -s yes -m intersection-strict -t gene -i ID) and the S288c R63-1-1 annotation file; an average of 44 M reads per strain mapped to annotated genes.

### Differential Expression Analysis

Differential expression analysis was performed using DESeq v1.10.1 (Anders and Huber 2010). Since there were no biological replicates, the “blind” method was used to estimate dispersions. This uses all samples to determine the typical variance in expression for each gene. Since the clones are derived from the same parental strain and contain only a few mutations, we believe this is an acceptable method to estimate dispersions. Differential expression was determined for all evolved clones against the diploid ancestral strain. Genes were considered differentially expressed between two strains if their adjusted *P*-value was less than 0.05. Complete DESeq output files are included in GEO Accession #GSE95069. RNA-seq and GO term analysis are summarized in the RNA-seq analysis table (Supplemental Materials online).

### qRT-PCR Analysis

RNA was reverse transcribed using Multiscribe reverse transcriptase (Thermo Fisher #4311235) with random hexamers. cDNA was diluted to 1:100 and quantified using targeted qPCR primers (Supplementary Materials online) and SYBR select (Life Technologies #4472908) on the Biorad CFX qPCR system. A standard curve was used to determine linear range and efficiency of the primers. Gene expression was internally normalized to ACT1 expression and error propagated for replicates (qPCR analysis, Supplementary Materials online). Unless otherwise noted, gene expression was measure in triplicate reactions for each of two biological duplicates. To the best of our ability, we attempted to process each of the ∼100 evolved clones and ancestral strains at the same time to prevent variation within a biological replicate.

### Cluster Analysis

All clustering analysis was performed on the log_2_-transformed qRT-PCR gene expression ratio between the evolved clones and the diploid ancestral strains internally normalized to *ACT1* expression. Hierarchical clustering analysis was performed using the R (v3.3.2, 2016-10-31) hclust function with the “complete” clustering method. Linear discriminate analysis was performed using the lda function in the MASS package (v7.3-45). LDA was performed twice, classifying the strains by either adaptive mutation or by ancestral ploidy (1N, 2N, or 4N).

## Supplementary Material

Supplementary data are available at *Molecular Biology and Evolution* online.

## Supplementary Material

Supplementary Data
