# Supplementary material for: The Influence of Polyploidy on the Evolution of Yeast Grown in a Sub-Optimal Carbon Source
Source: Mol Biol Evol. 2017 Jul 24;34(10):2690–703. doi: 10.1093/molbev/msx205 (PMC5850772; doi:10.1093/molbev/msx205)
Supplement: Supplementary Data [file msx205_Supp.zip › msx205_SuppFigandTables.pdf]

Supplementary Text 1 – Distribution of total mutations by ploidy level

We previously used whole genome sequencing (WGS) and comparative genome hybridization microarray (aCGH) to detect SNPs, indels and CNVs in 22 haploid evolved, 24 diploid evolved, and 28 tetraploid evolved clones (Selmecki et al. 2015). We reported that the 4N-evolved clones acquired significantly more mutations that the 1N-evolved and 2N-evolved clones. However, to understand the molecular mechanisms that lead to the adaptation of the 4N-evolved lineages to raffinose, and how these differed from 1N-evolved and 2N-evolved lineages, we further quantified the types of mutations gained in each of the evolved clones. Each genomic variant detected in the evolved clones was annotated by SNPeff (v4.1) as synonymous SNP, non-synonymous SNP, intragenic indel, intergenic SNP or intergenic indel (See Methods). The fraction of non-synonymous SNPs per ploidy level (21/31, 47/57, 71/89 in 1N-evolved, 2N-evolved, and 4N-evolved clones, respectively) is not significantly different from expectation, given that ~79% of mutations that occur in the coding region are expected to be non-synonymous (p=0.123, p=0.627, p=1 in the 1N-evolved, 2N-evolved, and 4N-evolved clones respectively, exact binomial test) (Wenger et al. 2011). Additionally, the number of intergenic mutations in the 2N-evolved (12/69, p=0.059 exact binomial test) and 4N-evolved (37/126, p=0.766 exact binomial test) clones is not significantly different than expectation, given that 72% of the genome is coding (Wenger et al. 2011). However, the 1N-evolved clones had fewer intergenic mutations than expected (1/45, $p=1.4x10^{-5}$ exact binomial test). Therefore, the distribution of mutations in the 1N-evolved clones is significantly different than the 2N-evolved ($p=2.71x10^{-6}$, fisher exact test) and 4N-evolved ($p=1.12x10^{-9}$, fisher exact test) clones (Supplementary Figure S1), suggesting differing mechanisms of adaptation in the 1N-evolved clones compared to the 2N-evolved and 4N-evolved clones.

Supplementary Text 2 – Review of glucose sensing pathway in yeast

Raffinose, a poor source of extracellular carbon, is hydrolyzed extracellularly by the invertase Suc2 into fructose and melibiose; while melibiose cannot be further utilized in our strain background, the fructose is metabolized equivalently to glucose by the glucose pathway in yeast (Naumov et al. 1990; Lagunas 1993). There are three key steps that lead to glucose uptake in yeast: the glucose sensors, signal transducers, and hexose transporters (Figure 1C). The glucose sensors, Snf3 and Rgt2*,* signal to downstream regulators of glucose-induced genes in the presence of low or high extracellular glucose concentrations, respectively (Ozcan et al. 1996; Sabina and Johnston 2009). Under glucose-depleted conditions, Mth1 binds Rgt1 and represses the transcription of glucose inducible genes, such as those encoding the hexose transporters and Suc2*.* (Kim et al. 2003; Polish et al. 2005; Roy et al. 2013). However, in the presence of low extracellular glucose concentrations, Snf3 signals the degradation of Mth1, resulting in Rgt1 phosphorylation and de-repression of glucose-inducible genes (Lafuente et al. 2000; Flick et al. 2003; Polish et al. 2005; Pasula et al. 2010). The hexose transporters Hxt1-Hxt7 transport glucose into the cell and differ in their regulation and affinity for glucose (Liang and Gaber 1996; Boles and Hollenberg 1997; Lin and Li 2011).

Supplementary Figures

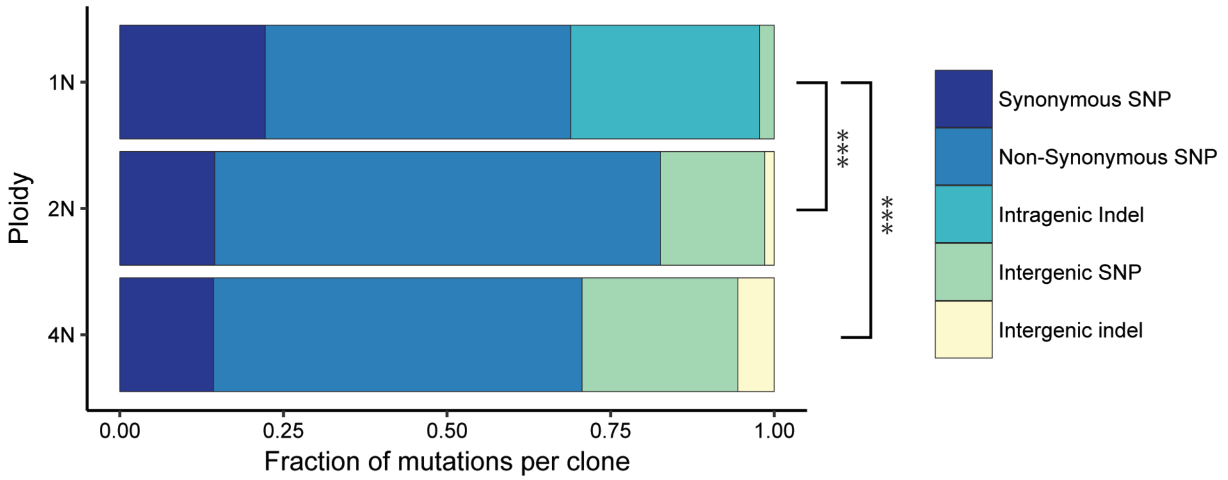

Supplementary Figure S1. The distribution of mutations in the evolved clones vary by the initial ploidy. The fraction of each mutation type in the 1N (n=22), 2N (n=24), and 4N (n=28) experimentally evolved clones. Mutations obtained from Selmecki et al. (2015) were categorized as synonymous SNP, non-synonymous SNP, intragenic indel, intergenic SNP or intergenic indel. The distribution of total mutations is significantly different in the 1N evolved clones compared to the 2N ($p=2.71x10^{-6}$) and 4N ($p=1.12x10^{-9}$) evolved clones. Significance was determined by the fisher exact test for count data, corrected for multiple hypothesis testing.

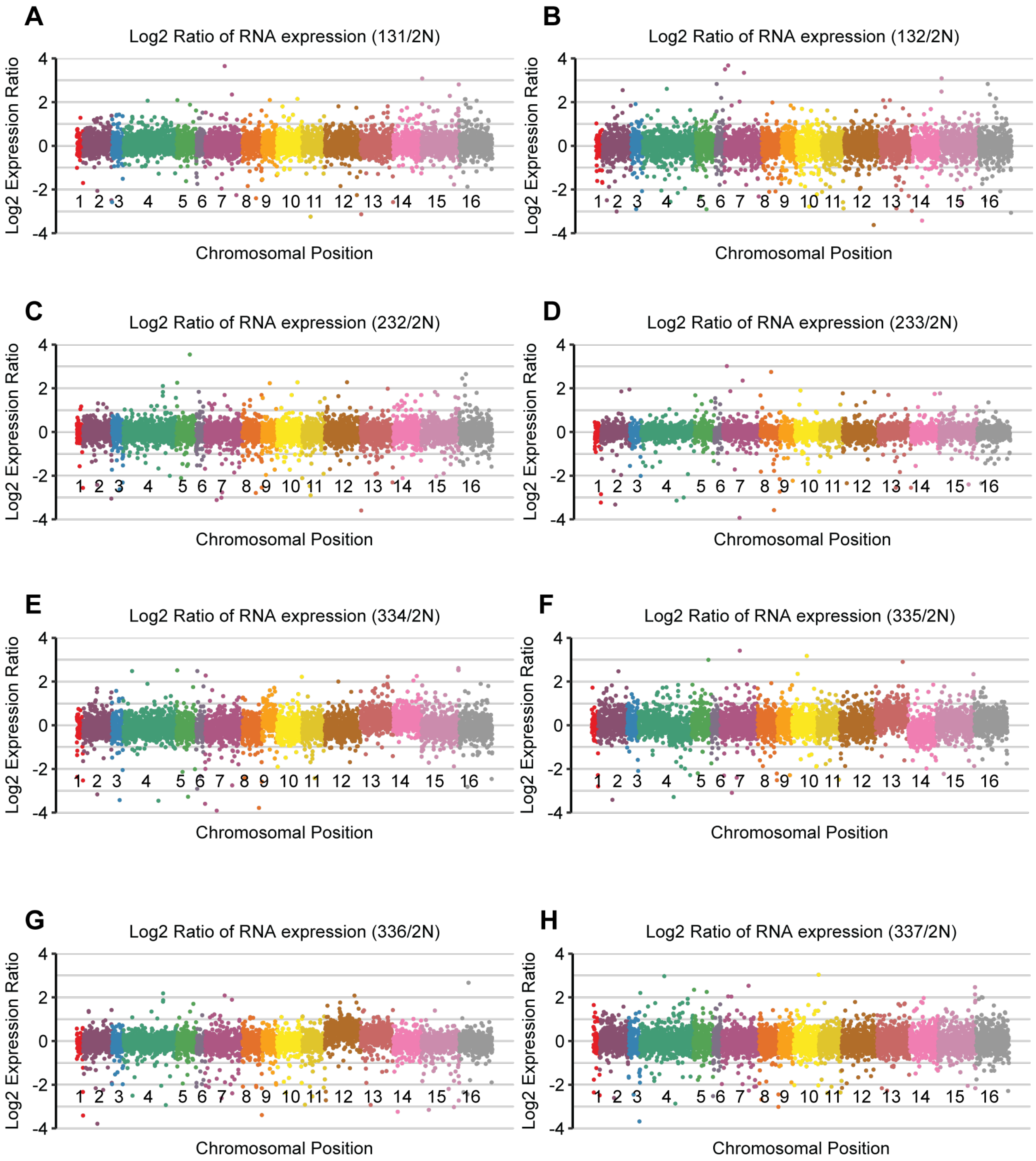

Supplementary Figure S2. Quantification of mRNA abundance levels in the evolved clones. Log_2-transformed RNA expression ratio in the following strains relative to the diploid ancestral strain: A) 131, B) 132, C) 232, D) 233, E) 334, F) 335, G) 336, and F) 337. All strains were grown in 2% raffinose medium and each dot represents the expression ratio (y-axis) of a single gene plotted with respect to their chromosome position (x-axis). All evolved clones are euploid and of their ancestral ploidy, except 4N-evolved clones 334, 335 and 336. Clone 334 is

near-triploid with tetrasomic copies of chromosomes (Chr) 9, 13, and 14; Clone 335 is near-tetraploid with a segmental deletion of Chr4, pentasomic Chr13, and trisomic Chr14; clone 336 is tetraploid with hexasomic Chr12, and pentasomic Chr13 (Selmecki et al. 2015).

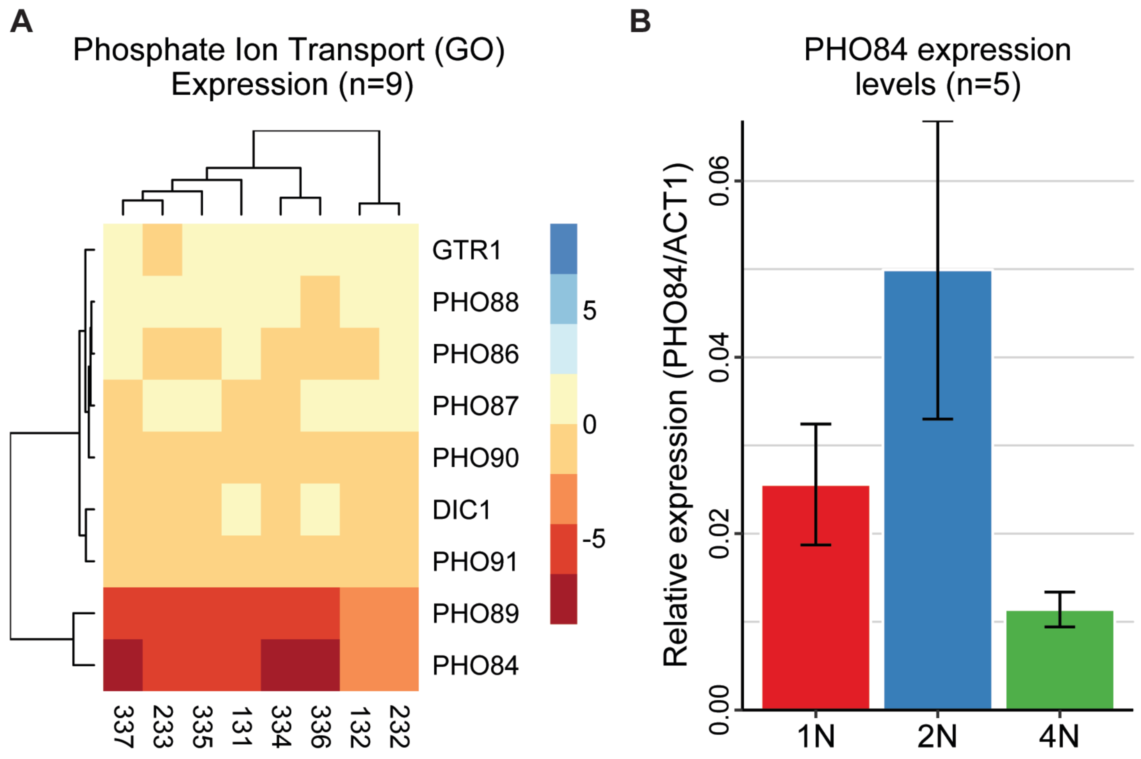

Supplementary Figure S3. Evolved clones differentially regulate phosphate ion transport. A) Log_2-transformed expression of genes annotated with the GO-function of phosphate ion transport. Phosphate ion transport is decreased in all of the evolved clones; primarily driven by the overexpression of *PHO84* and *PHO89* in the diploid ancestor. B) qRT-PCR expression of *PHO84* relative to *ACT1* expression in the haploid (red), diploid (blue), and tetraploid (green) of 5 biological replicates. Error bars represent the standard error of the mean (SEM). Only the diploid ancestor consistently had elevated *PHO84,* though with high variability. Regulation of the phosphate genes is controlled by a hysteretic switch that may contribute to variability in activation of the phosphate transporters (Raser and Shea 2006).

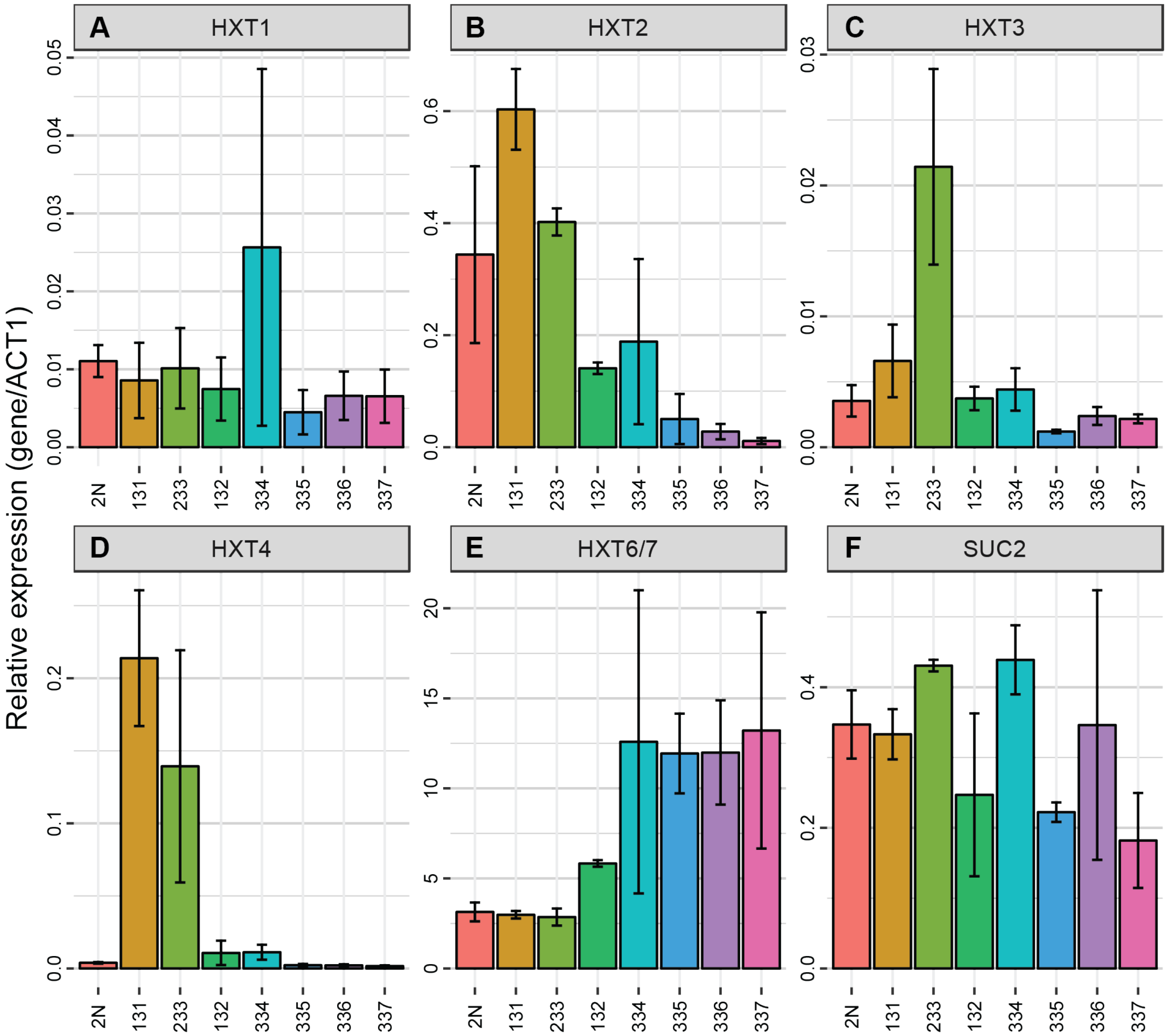

Supplementary Figure S4. Confirmation of hexose transporter expression in the evolved clones by qRT-PCR. qRT-PCR gene expression analysis of glucose responsive genes in the diploid ancestor and evolved clones grown in 2% raffinose medium. All genes are normalized to *ACT1* expression. A) *HXT1*, B) *HXT2*, C) *HXT3*, D) *HXT4*, E) *HXT6/7*, and F) *SUC2*. n=2, error bars represent SEM.

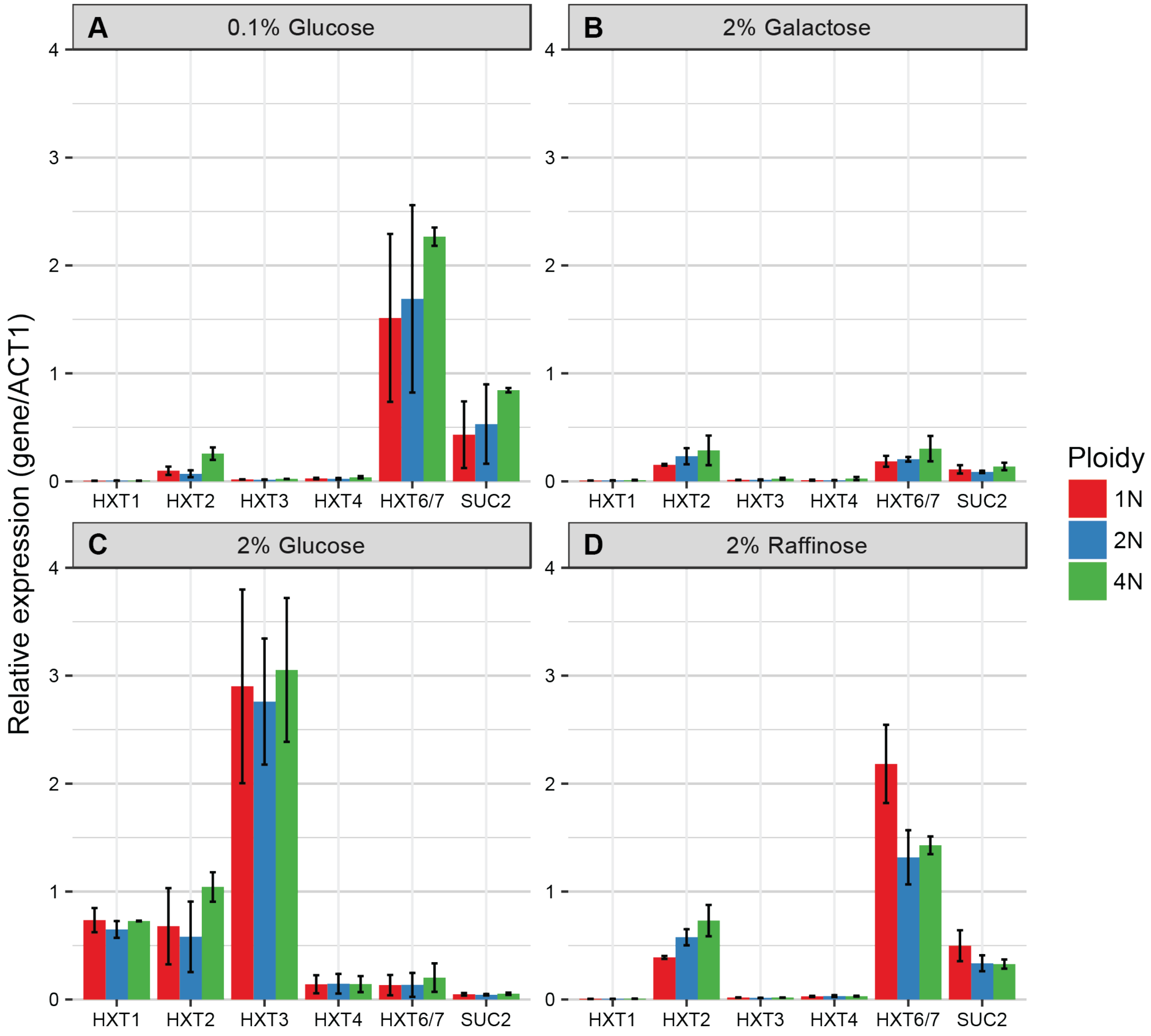

Supplementary Figure S5. Ancestral ploidy does not alter the expression of glucose responsive genes in various carbon sources. qRT-PCR gene expression analysis of glucose responsive genes (*HXT1, HXT2, HXT3, HXT4, HXT6/7*, and *SUC2* internally normalized to *ACT1* expression for the 1N, 2N, and 4N ancestral strains grown in A) 0.1% Glucose, B) 2% Galactose, C) 2% Glucose, and D) 2% Raffinose. n=2, error bars represent SEM.

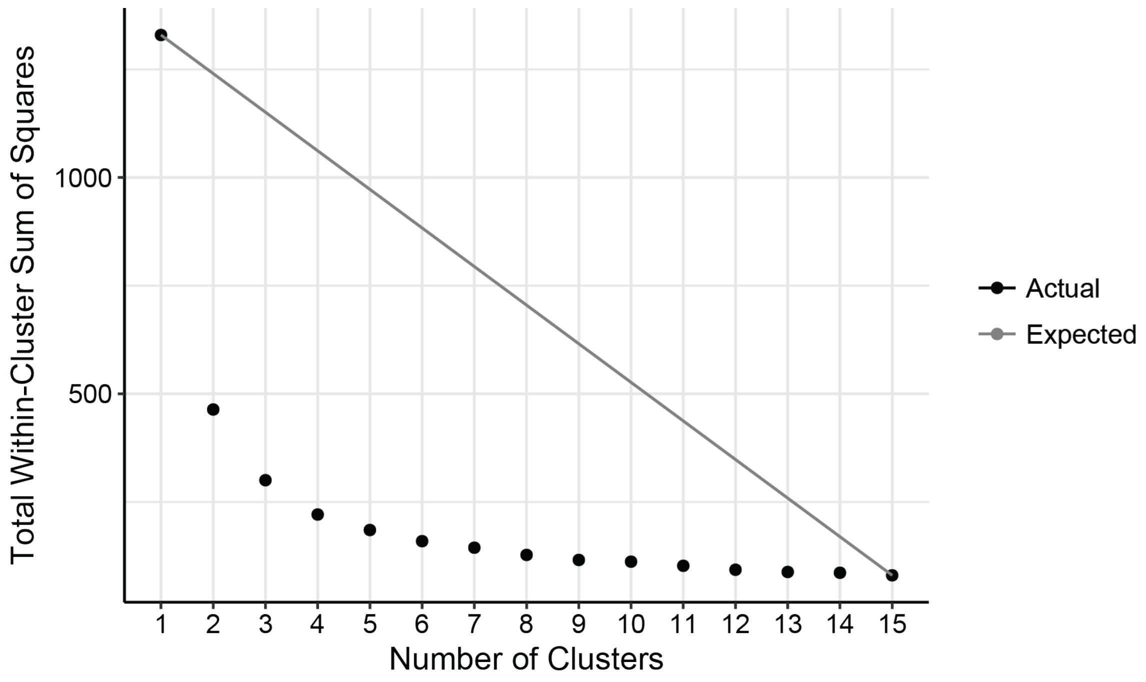

Supplementary Figure S6. Within group variance is minimized at k=5. The averages total sum of squares (TSS) (Y-axis) for k clusters (x axis) was quantified using k-means for the $\log_2$-transformed gene panel expression data. The TSS was averaged over 20 trials (black dots) at each k. The null hypothesis (grey line) is that the variance decreases linearly with model complexity. The optimal number of groups was determined by visual inspection of the number of clusters (k) at which the change in TSS becomes linear.

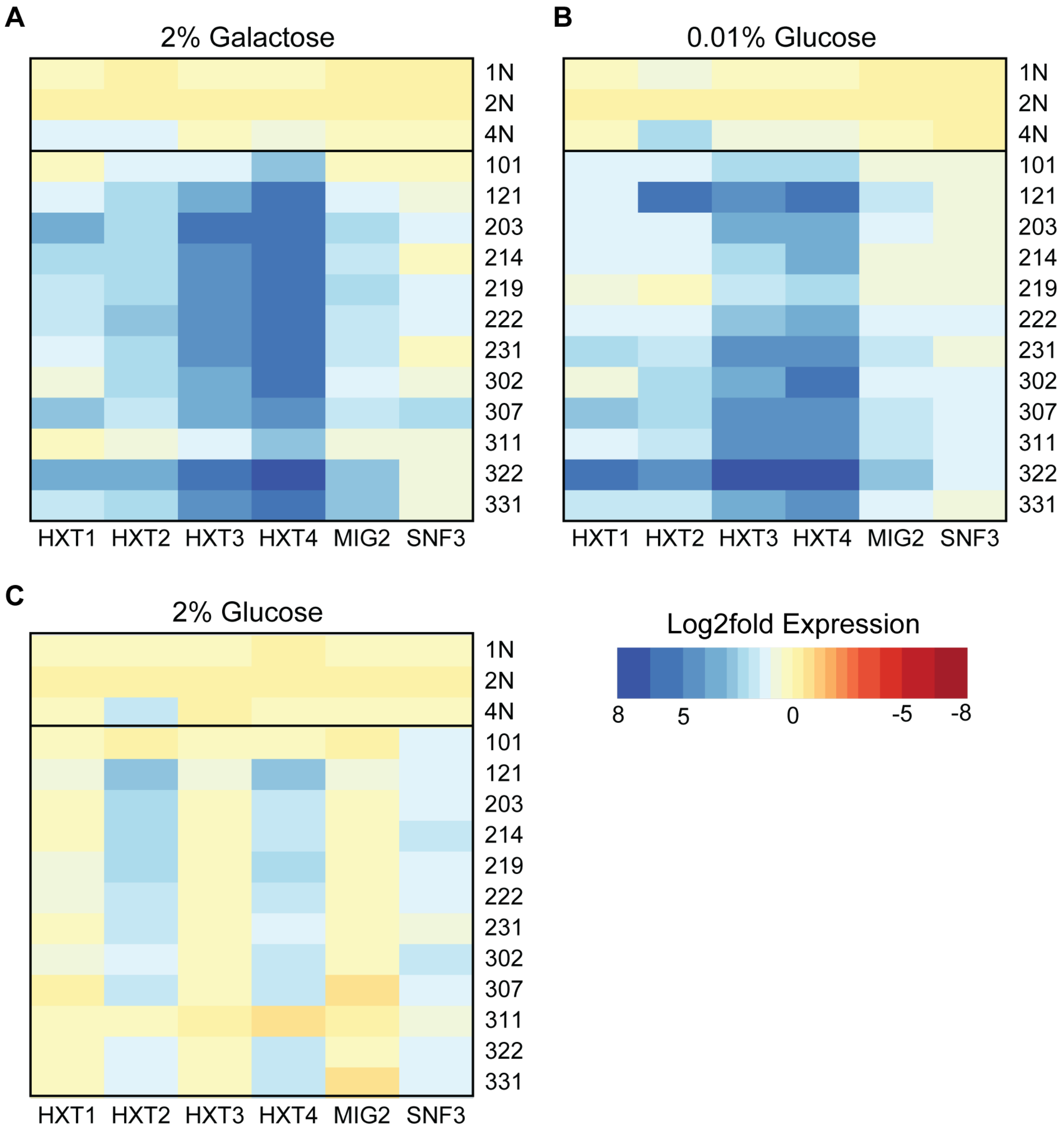

Supplementary Figure S7. Mutations in SNF3 result in constitutive activation of glucose responsive genes. qRT-PCR expression analysis of glucose responsive genes normalized to *ACT1* for selected evolved clones with mutations in *SNF3* relative to the diploid ancestral strain. Strains were grown 0.1% glucose overnight and then grown in A) 2% Galactose, B) 0.1% Glucose, and C) 2% Glucose for 6 hours or until cultures reached an OD of 0.8.

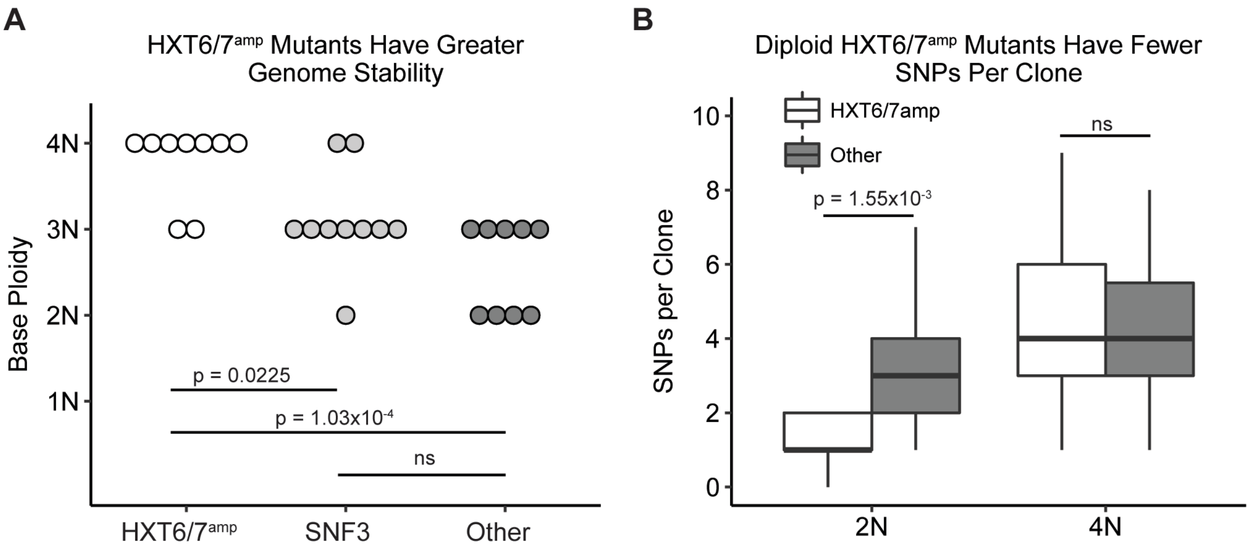

Supplementary Figure S8. Evolved clones with amplification of the *HXT6/7* region acquire fewer mutations and remain primarily tetraploid. A) Base ploidy level of the 4Ne clones with *HXT6/7* amplifications, *SNF3* mutations, or other mutations. Clones with *HXT6/7^{amp}* have a significantly higher ploidy level than *SNF3* mutants ($p=2.25\times10^{-2}$, ANOVA with Tukey post-hoc test, df = 2) and clones with other mutations ($p=1.03\times10^{-4}$). There is no significant difference between clones with mutations in *SNF3* compared to clones with mutations in other genes. The base ploidy level was determined with flow cytometry analysis for DNA content aCGH or WGS as previously reported (Selmecki et al. 2015). B) The average number of SNPs per strain in the 2N-evolved clones and 4N-evolved clones with HXT6/7^{amp} (white) or without HXT6/7^{amp} (grey). The 2N-evolved clones with HXT6/7^{amp} have significantly fewer SNPs per strain than the clones with only a single copy of *HXT6* and *HXT7* ($p=1.55\times10^{-3}$, Welch two sample t-test, df=13.473).

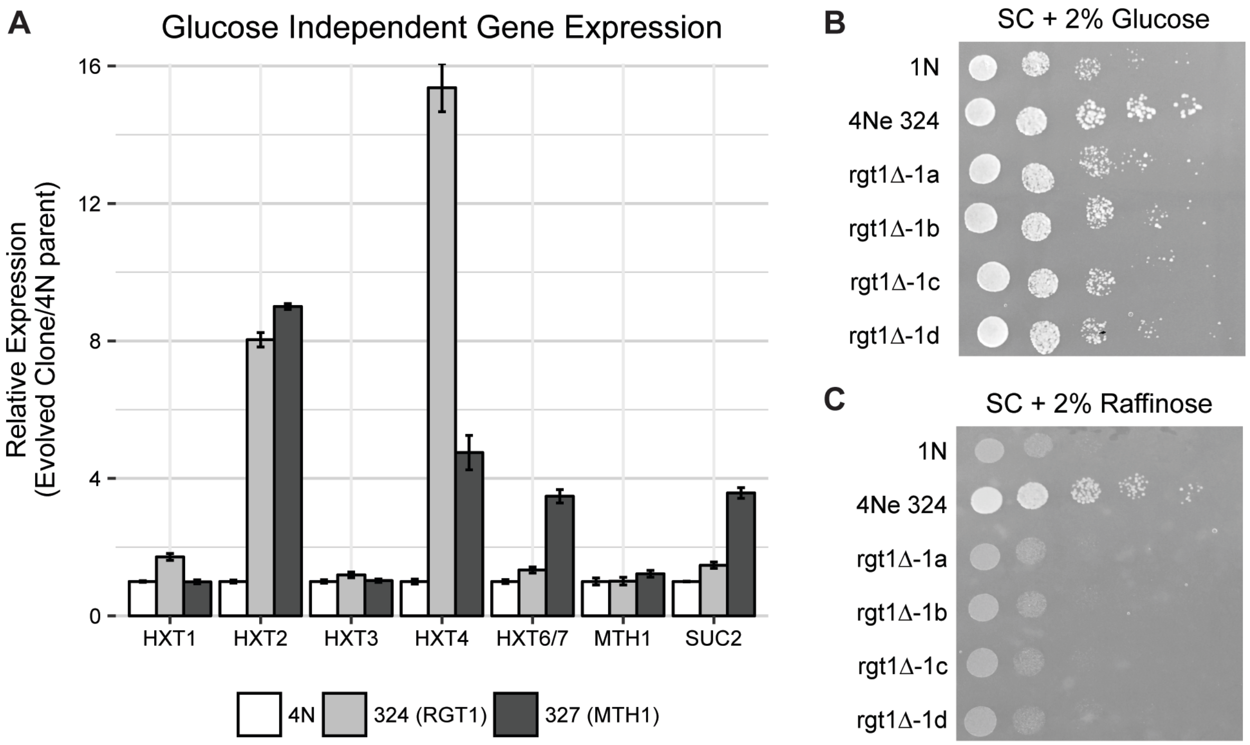

Supplementary Figure S9. Characterization of *RGT1* and *MTH1* dominant mutations. A) qRT-PCR gene expression analysis of glucose responsive genes relative to *ACT1* expression and normalized to the 4N ancestral strain grown in 2% galactose. Spot assay for growth of *RGT1* deletion strains grown on SC plates with (B) 2% Glucose or (C) 2% Raffinose. Spot assays were performed by growing strains to saturation in YPD or the corresponding selective medium for *rgt1Δ* (YPD + 200μg/mL G418) or 4N-evolved clone 324 (SC+2% raffinose). Cells were washed and re-suspended in water to a density of 1 ODU/ml. Cells were 10-fold serially diluted in water and 10ul was spotted on SC plates containing either 2% glucose or 2% raffinose. Plates were incubated at 30°C for 72 hours before photographing.

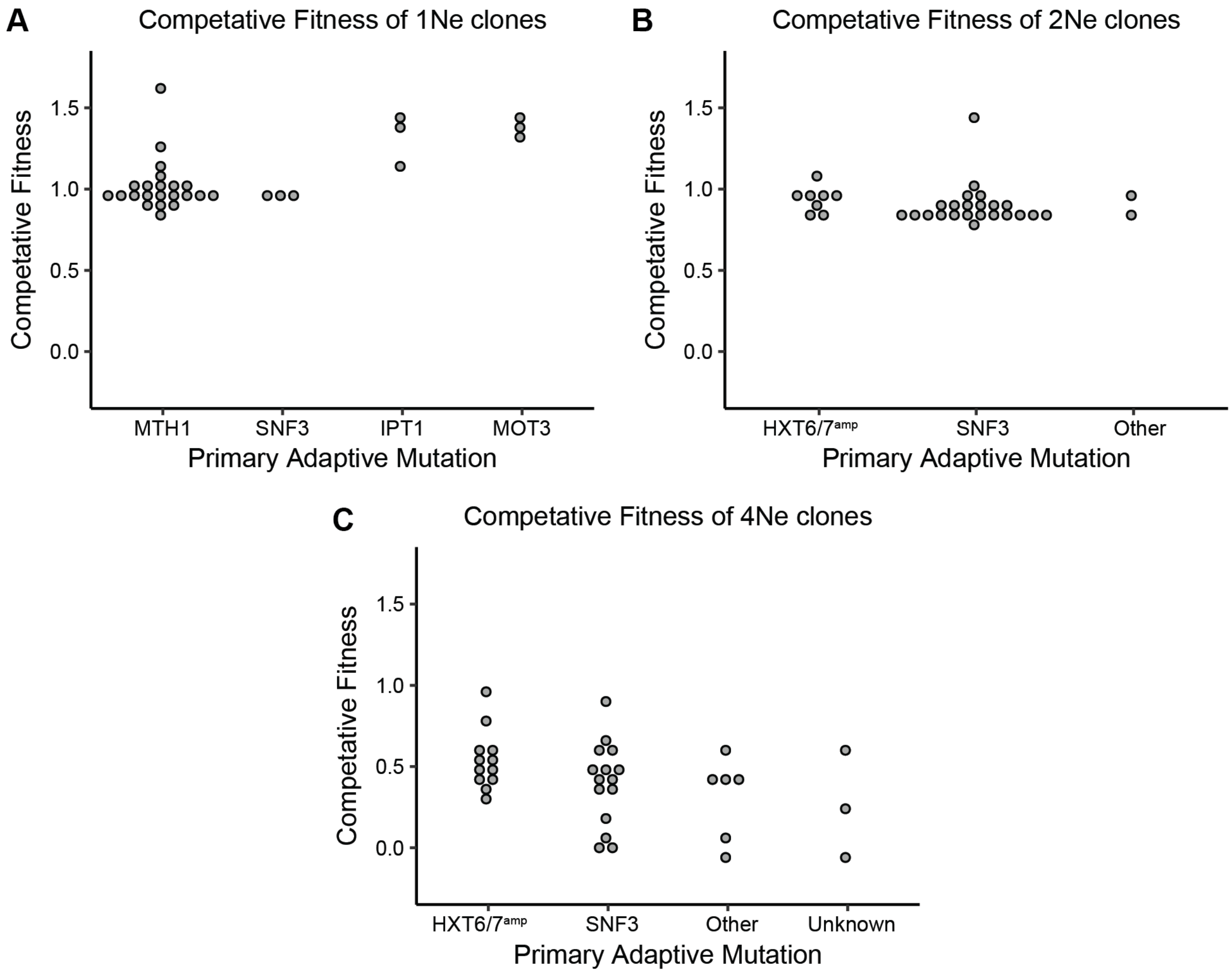

Supplementary Figure S10. Evolved clones show dramatic fitness improvements over ancestral strains. Competitive fitness of the evolved clones isolated from A) 1N, B) 2N, C) and 4N populations. Competitive fitness (Y-axis) is relative to the 2N ancestral strain as described in (Selmecki et al. 2015), where the fitness of the 2N ancestor is set to zero. The evolved clones are separated by primary adaptive mutation (X-axis).

Supplementary Tables

Strain/ Clone	Initial Ploidy Level	Total # of mutations	Adaptive Mutation(s)	HXT6/7 Status	Other Mutations
**DP5999**	1N (ancestor)	NA	NA	NA	NA
**DP6006**	2N (ancestor)	NA	NA	NA	NA
**DP6040**	4N (ancestor)	NA	NA	NA	NA
**101**	1N	2	SNF3 (G519C)	qPCR No HXT	YPR130C(T56M)
**102**	1N	1	MTH1 (I353fs)	qPCR No HXT	none
**103**	1N	1	IPT1 (C219W)	qPCR No HXT	none
**104**	1N	3	MTH1 (I353fs)	CGH No HXT	MUK1(ns), YML018C (s)
**105**	1N	6	MTH1 (N12fs)	qPCR No HXT	YOL098C(N836Y), HRD1(W123Stop), MTH1(s), NUP1(s), PDR1(s)
**106**	1N	2	MTH1 (I353fs)	qPCR No HXT	YHR131C(s)
**108**	1N	2	MTH1 (I353fs)	qPCR No HXT	WHI3(s)
**109**	1N	1	MTH1 (K333Stop)	qPCR No HXT	none
**110**	1N	1	MTH1 (N69fs)	CGH No HXT	none
**111**	1N	1	MTH1 (K367fs)	qPCR No HXT	none
**112**	1N	3	MTH1 (S133fs)	qPCR No HXT	PXA2(G520A), FMP27(s)
**113**	1N	1	MOT3 (R357Stop)	CGH No HXT	none
**114**	1N	1	MOT3 (P268fs)	CGH No HXT	none
**115***	1N	unknown	MTH1 (I353fs)	qPCR No HXT	
**116**	1N	1	MTH1 (I353fs)	qPCR No HXT	none
**117**	1N	1	IPT1 (V232D)	qPCR No HXT	none
**118**	1N	2	SNF3 (A488D)	qPCR No HXT	YIL025C(R124Q)
**119***	1N	unknown	MTH1 (I353fs)	qPCR No HXT	
**120**	1N	3	MOT3 (K394Stop)	qPCR No HXT	LOH1(T201K), MKK2(R455T)
**121**	1N	2	SNF3 (V470D)	qPCR No HXT	FAS1 (s)
**122**	1N	1	MTH1 (I353fs)	qPCR No HXT	none
**124**	1N	4	MTH1 (I353fs)	qPCR No HXT	ACA1(Y36N), YIL102C-A(S40R), NCS6(s)
**126***	1N	unknown	MTH1 (S133fs)	qPCR No HXT	
**127**	1N	4	MTH1 (A275D)	qPCR No HXT	THO2(S1496P), MAK5 (s), chrX 29781
**128**	1N	2	MTH1 (I353fs)	qPCR No HXT	KAR5(F197I)
**131$^{\Phi}$**	1N	5	MTH1 (I353fs)	CGH No HXT	NSE4(G93D), HRD3(T436Stop), RAD33(s), TRM732(S626N)
**132$^{\Phi}$**	1N	4	IPT1(Y15Stop) & HXT6/7^{amp}	CGH +HXT	DPB3(D128G), YLH47(P263Q)

**201***	2N	unknown	HXT6/7^{amp}	CGH +HXT	
**202**	2N	3	SNF3(G439R)	qPCR No HXT	YGL039W(S38P), ChrIV-1019186
**203**	2N	1	SNF3(D114Y)	qPCR No HXT	none
**204**	2N	5	RGT2(S215F)	qPCR No HXT	SLA1(Q885E), IRA1(s), SNX4(s), PHO80(s)
**205**	2N	1	HXT6/7^{amp}	qPCR + HXT	POS5(N413D)
**206**	2N	3	SNF3(D114Y)	qPCR No HXT	MCM3(K759M), MED4(P65Q)
**207**	2N	2	SNF3(F458L) & HXT6/7^{amp}	qPCR + HXT	RTK1(D59H)
**208**	2N	3	SNF3(Q220E)	qPCR No HXT	SGF73(E356G), ChrXV-544522
**209**	2N	2	SNF3(D114Y)	qPCR No HXT	AZF1(s)
**210**	2N	2	SNF3(G101A)	qPCR No HXT	EMP65(s)
**211**	2N	3	SNF3(D114Y)	qPCR No HXT	AIM14(I21F), UBP13(V590F)
**212**	2N	0	HXT6/7^{amp}	CGH +HXT	none
**213**	2N	2	SNF3(G101A)	qPCR No HXT	ChrXIII-499722
**214**	2N	3	SNF3(A214E)	qPCR No HXT	VPS27(H243N), chrXII-950618
**215***	2N	unknown	SNF3(A214E)	qPCR No HXT	
**216***	2N	unknown	SNF3(G157A)	qPCR No HXT	
**217***	2N	unknown	HXT6/7^{amp}	CGH +HXT	
**218**	2N	2	HXT6/7^{amp}	CGH +HXT	YKL133C(Y287Stop), AVO2(C124F)
**219**	2N	2	SNF3(F458L)	qPCR No HXT	chrIV-771695
**220***	2N	unknown	SNF3(G157A)	qPCR No HXT	
**221**	2N	4	SNF3(D114Y)	qPCR No HXT	MDM31(F121C), SPS1(s), chrVIII-48667
**222**	2N	4	SNF3(R417T)	qPCR No HXT	BCS1(L41V), PDR12(G678R), chrXIII-237806
**223**	2N	7	SNF3(F111L)	qPCR No HXT	BRN1(A556V), SEC7(P706A), YBT1(s), NOP58(s), chrX-153262, chrXI-161378
**224**	2N	5	SNF3(D114Y)	qPCR No HXT	SMC1(Q294Stop), HIF1(D370Y), YOR389W(E203Stop), BNI1(s)
**225**	2N	2	SNF3(D114Y)	qPCR No HXT	SWA2(S362F)
**226**	2N	3	SNF3(D114Y)	qPCR No HXT	MCM3(F520C), BIR1(N718H)
**227**	2N	7	SNF3(F458L)	qPCR No HXT	UBP1(S670P), VID30(Y20D), INO1(A282V), NIP100(s), chrXV-326628, chrXV-581532
**228***	2N	unknown	HXT6/7^{amp}	CGH +HXT	
**229***	2N	unknown	SNF3(G439E)	qPCR No HXT	
**230***	2N	unknown	HXT6/7^{amp}	CGH +HXT	
**231***	2N	unknown	SNF3(V470F)	qPCR No HXT	
**232**	2N	1	HXT6/7^{amp}	CGH +HXT	ChrXVII-746160
**233**	2N	2	SNF3(G439E)	CGH No HXT	LTE1(S626C)

**301***	4N	unknown	SNF3(T385R)	CGHed No HXT	
**302***	4N	unknown	SNF3(E550K)	CGHed No HXT	
**303***	4N	unknown	SNF3(A491D)	qPCR No HXT	
**304**	4N	6	SNF3(G439V)	CGHed No HXT	YKL222C(S697T), UTR2 (s), chrXIII-23797, chrXIII-653210, chrXV-106555
**305**	4N	8	SNF3(E413K)	CGHed No HXT	HBT1(E938G), RPN4(T259M), ALR2(G529R), FAP1(Q474H), NEW1(S277Y), EIS1 (s), chrXVI-215384
**306**	4N		Unknown	qPCR No HXT	
**307**	4N	3	SNF3(R229K)	qPCR No HXT	FAS2(G498V), GDE1(A1212V)
**308***	4N	unknown	SNF3(A491D)	qPCR No HXT	
**309**	4N	5	SNF3(T385R)	qPCR No HXT	MAK32(M1L), SCH9(S104P), IMD2(P130T), YDR161W (s)
**310**	4N	7	SNF3(G230S)	CGHed NoHXT	FMS1(H131N), VMR1 (s), CHC1 (s), chrXIII-463918, chrXIII-756312, chrXIV-380225
**311**	4N	5	SNF3(T224R)	qPCR No HXT	COX18(M65L), SLN1(G769R), ALP1 (s), chrVI-15959
**312**	4N	1	$HXT6/7^{amp}$	qPCR +HXT	
**313**	4N	8	SNF3(G349V)	CGHed No HXT	LHS1(G201V), ALD5(N170I), CDC31(N152K), UTR2 (s), chrIV-488849, chrXIII-23797, chrXIII-653210
**314**	4N	8	Unknown	qPCR No HXT	RAX1(F14L), IOC2(M554I), MSC7(V245F), YLR345W(W109S), ChrIII-84937, ChrIV-229797, ChrVI-256200, ChrIX-277539
**315**	4N	4	RGT2(G418D)	CGHed No HXT	SPP41(R147Q), LCB5(E504K), ALR2 (s)
**316**	4N	1	RGT2(D415N)	CGHed No HXT	none
**317***	4N	unknown	$HXT6/7^{amp}$	CGHed +HXT	
**318***	4N	unknown	SNF3(E413K)	CGHed No HXT	
**319**	4N	3	$HXT6/7^{amp}$	CGHed +HXT	GEP5 (s), ChrVIII-488371, ChrXIII-211720
**320**	4N	4	$HXT6/7^{amp}$	CGHed +HXT	XRN1(L1078I), ChrIV-1154143, ChrIV-1154155, ChrIV-1154200
**321**	4N	3	Unknown	CGHed No HXT	PCS60(P267S), DUR3(G465C), ChrIX-277539
**322**	4N	5	SNF3(G468R)	qPCR No HXT	ORC4(P10L), SET6 (s), chrVII-328015, chrXV-1013114
**323**	4N	4	SNF3(T224R)	qPCR No HXT	PRS4(I137M), SSS1(A4T), chrVXII-856265
**324**	4N	3	RGT1 (S509stop)	qPCR No HXT	TDH1(C154S), SOF1(P121A)
**325***	4N	unknown	$HXT6/7^{amp}$	qPCR +HXT	

**326***	4N	unknown	HXT6/7^{amp}	qPCR +HXT	
**327**	4N	2	MTH1(C321F)	qPCR No HXT	BUD27(A641S)
**328**	4N	6	HXT6/7^{amp}	qPCR +HXT	HCA4(G382R), SMC4(L673Stop), YPL264C(V57G), SPT1 (s), HXT7 (s), ChrII-804908
**329**	4N	5	Unknown	CGHed No HXT	TOR2(M488I), RPA190(V266I), YGR168C(R318W), SPT15 (s), ChrII-278810
**330***	4N	unknown	HXT6/7^{amp}	CGHed +HXT	
**331**	4N	3	SNF3(V470F)	qPCR No HXT	ATS1 (s), HHO1 (s)
**332**	4N	9	HXT6/7^{amp}	qPCR +HXT	CHD1(ns), COG1(T88I), SRP102(K155Q), GDE1(D1203A), ChrIV-115413, ChrIV-1154155, ChrIV-154200, ChrVI-243118
**333**	4N	3	HXT6/7^{amp}	qPCR +HXT	NEW1(F321V), YCL041C (s), ChrVIII-234713
**334**	4N	3	Unknown	CGHed No HXT	TOR2(M488I), YTA7(M710L), SPT15 (s)
**335**	4N	6	HXT6/7^{amp}	CGHed +HXT	PDC2(V138I), VTC2(A729S), YSP3(s), TFB4(Q17K), EFM1(1550L), YGR266W(A411T)
**336**	4N	3	HXT6/7^{amp}	CGHed +HXT	PBP1(F380Y), FAS1(E1026D), ChrVIII-375275
**337**	4N	5	HXT6/7^{amp}	CGHed +HXT	PGU1(I17N), DIN7(T90S), SNG1(A507V), SPG5 (Q175Stop), ChrII-289178

Supplementary Table S1. Summary of mutations identified in the evolved clones utilized in this study. The identity of the adaptive mutations and other mutations identified in the evolved clones utilized in this study. Clones for which there is no whole genome sequencing available are denoted with an asterisk (*). Clones sequenced in this study are denoted with (ϕ), all other clones were sequenced in Selmecki et al. (2015). Intragenic mutations classified as synonymous (s), non-synonymous, or frameshift (fs). The amino acid substitution is listed after the gene name for mutations classified as non-synonymous. Intergenic mutations are listed by the chromosome coordinate.

Clone	Clade	SNF3 mutation	TMHMM	Ploidy Level
**118**	*a*	A488D	TM11	1N
**303***	*a*	A491D	TM11	4N-
**308***	*a*	A491D	TM11	4N-
**203**	*a*	D114Y	TM1	2N
**206**	*a*	D114Y	TM1	2N
**318***	*a*	E413K	TM8	2N
**322**	*a*	G468R	TM10	3N
**121**	*a*	V470D	TM10	1N
**214**	*b*	A214E	TM4	2N
**215***	*b*	A214E	TM4	2N
**209**	*b*	D114Y	TM1	2N
**211**	*b*	D114Y	TM1	2N
**221**	*b*	D114Y	TM1	2N
**224**	*b*	D114Y	TM1	2N
**225**	*b*	D114Y	TM1	2N
**226**	*b*	D114Y	TM1	2N
**302***	*b*	E550K	C-TAIL	3N+/-
**223**	*b*	F111L	TM1	2N
**216***	*b*	G157A	TM2	2N
**220***	*b*	G157A	TM2	2N
**233**	*b*	G439R	TM9	2N
**202**	*b*	G439R	TM9	2N
**101**	*b*	G519C	EC	1N
**208**	*b*	Q220E	TM4	2N
**307**	*b*	R229K	TM5	4N
**331**	*b*	V470F	TM10	2N
**231***	*b*	V470F	TM10	2N
**305**	*c*	E413K	TM8	3N+
**219**	*c*	F458L	TM10	2N
**227**	*c*	F458L	TM10	2N
**210**	*c*	G101A	TM1	2N
**213**	*c*	G101A	TM1	2N
**310**	*c*	G230S	TM5	3N
**229***	*c*	G439E	TM9	2N
**304**	*c*	G439V	TM9	3N+
**313**	*c*	G439V	TM9	3N
**222**	*c*	R417T	CYTO	2N
**311**	*c*	T224R	CYTO	4N
**323**	*c*	T224R	CYTO	4N
**309**	*c*	T385R	EC	4N-
**301***	d	T385R	EC	4N

Supplementary Table S2. Identity of *SNF3* mutations grouped by downstream gene activation. The identity of the amino acid substitutions in *SNF3* mutant strains profiled for

glucose responsive gene expression. The locations of transmembrane, cytosolic, and extracellular domains were predicted with TMHMM 2.0c (Krogh et al. 2001). Mutations are diagramed in Figure 3. The ploidy level was determined with flow cytometry analysis for DNA content aCGH or WGS as previously reported (Selmecki et al. 2015).

Mutation	Background	Strain	Experimental details	Citation
**H154Tfs**	W303 (1N)	EvoClone 2	1mM Sucrose, serial passage, POL3-L523D mutant	Koschwanez et al. 2013
**S51Ifs**	W303 (1N)	EvoClone 6	1mM Sucrose, serial passage, POL3-L523D mutant	Koschwanez et al. 2013
**W427C**	S288C (1N)	E1, Bulk	0.08% Glucose, Chemostat, 448 generations	Kvitek and Sherlock 2013
**Q416K**	S288C (1N)	E1, Bulk	0.08% Glucose, Chemostat, 448 generations	Kvitek and Sherlock 2013
**Y332Stop**	S288C (1N)	E1, Bulk	0.08% Glucose, Chemostat, 448 generations	Kvitek and Sherlock 2013
**C321W**	S288C (1N)	E1, Bulk	0.08% Glucose, Chemostat, 448 generations	Kvitek and Sherlock 2013
**Y311Stop**	S288C (1N)	E1, Bulk	0.08% Glucose, Chemostat, 448 generations	Kvitek and Sherlock 2013
**Y245Stop**	S288C (1N)	E1, Bulk	0.08% Glucose, Chemostat, 448 generations	Kvitek and Sherlock 2013
**Q236Stop**	S288C (1N)	E1, Bulk	0.08% Glucose, Chemostat, 448 generations	Kvitek and Sherlock 2013
**L156Stop**	S288C (1N)	E1, Bulk	0.08% Glucose, Chemostat, 448 generations	Kvitek and Sherlock 2013
**S106Stop**	S288C (1N)	E1, Bulk	0.08% Glucose, Chemostat, 448 generations	Kvitek and Sherlock 2013
**N314H**	S288C (1N)	E2, Bulk	0.08% Glucose, Chemostat, 448 generations	Kvitek and Sherlock 2013
**R385Stop**	S288C (1N)	E2, Bulk	0.08% Glucose, Chemostat, 448 generations	Kvitek and Sherlock 2013
**E249Stop**	S288C (1N)	E2, Bulk	0.08% Glucose, Chemostat, 448 generations	Kvitek and Sherlock 2013
**C188Stop**	S288C (1N)	E2, Bulk	0.08% Glucose, Chemostat, 448 generations	Kvitek and Sherlock 2013
**S101Stop**	S288C (1N)	E2, Bulk	0.08% Glucose, Chemostat, 448 generations	Kvitek and Sherlock 2013
**K86Stop**	S288C (1N)	E2, Bulk	0.08% Glucose, Chemostat, 448 generations	Kvitek and Sherlock 2013
**Q374Stop**	S288C (1N)	E3, Bulk	0.08% Glucose, Chemostat, 448 generations	Kvitek and Sherlock 2013
**Q338Stop**	S288C (1N)	E3, Bulk	0.08% Glucose, Chemostat, 448 generations	Kvitek and Sherlock 2013
**L241Stop**	S288C (1N)	E3, Bulk	0.08% Glucose, Chemostat, 448 generations	Kvitek and Sherlock 2013
**S209Stop**	S288C (1N)	E3, Bulk	0.08% Glucose, Chemostat, 448 generations	Kvitek and Sherlock 2013
**1353fs**	S288C (1N)	102	2% Raffinose, serial passage, 250 generations	Selmecki et al. 2015

**1353fs**	S288C (1N)	104	2% Raffinose, serial passage, 250 generations	Selmecki et al. 2015
**N12fs**	S288C (1N)	105	2% Raffinose, serial passage, 250 generations	Selmecki et al. 2015
**1353fs**	S288C (1N)	106	2% Raffinose, serial passage, 250 generations	Selmecki et al. 2015
**1353fs**	S288C (1N)	108	2% Raffinose, serial passage, 250 generations	Selmecki et al. 2015
**K333Stop**	S288C (1N)	109	2% Raffinose, serial passage, 250 generations	Selmecki et al. 2015
**N69fs**	S288C (1N)	110	2% Raffinose, serial passage, 250 generations	Selmecki et al. 2015
**K367fs**	S288C (1N)	111	2% Raffinose, serial passage, 250 generations	Selmecki et al. 2015
**S133fs**	S288C (1N)	112	2% Raffinose, serial passage, 250 generations	Selmecki et al. 2015
**1353fs**	S288C (1N)	116	2% Raffinose, serial passage, 250 generations	Selmecki et al. 2015
**1353fs**	S288C (1N)	122	2% Raffinose, serial passage, 250 generations	Selmecki et al. 2015
**1353fs**	S288C (1N)	124	2% Raffinose, serial passage, 250 generations	Selmecki et al. 2015
**A475D**	S288C (1N)	127	2% Raffinose, serial passage, 250 generations	Selmecki et al. 2015
**1353fs**	S288C (1N)	128	2% Raffinose, serial passage, 250 generations	Selmecki et al. 2015
**1353fs**	S288C (1N)	131	2% Raffinose, serial passage, 250 generations	Selmecki et al. 2015
**C321F**	S288C (4N)	327	2% Raffinose, serial passage, 250 generations	Selmecki et al. 2015
**1353fs**	S288C (1N)	115	2% Raffinose, serial passage, 250 generations	Current Study
**1353fs**	S288C (1N)	119	2% Raffinose, serial passage, 250 generations	Current Study
**S133fs**	S288C (1N)	126	2% Raffinose, serial passage, 250 generations	Current Study

Supplementary Table S3. *MTH1* mutations recovered from yeast populations evolved in low glucose medium. We performed a literature search to find other reported mutations in *MTH1* obtained from experimental evolution in carbon-limited conditions. Mutations are diagrammed in Figure 4A.

Mutation	Background	Strain	Expermintal details	Citation
**L832fs**	W303 (1N)	EvoClone4	1mM Sucrose, serial passage, POL3-L523D mutant	Koschwanez et al. 2013
**Q1053Stop**	W303 (1N)	EvoClone1	1mM Sucrose, serial passage, POL3-L523D mutant	Koschwanez et al. 2013
**G687V**	W303 (1N)	EvoClone 10	1mM Sucrose, serial passage, POL3-L523D mutant	Koschwanez et al. 2013
**Y526C**	S288C (1N)	E1, Bulk	0.08% Glucose, Chemostat, 448 generations	Kvitek and Sherlock pgen 2013
**V565L**	S288C (1N)	E1, Bulk	0.08% Glucose, Chemostat, 448 generations	Kvitek and Sherlock 2013
**L575W**	S288C (1N)	E1, Bulk	0.08% Glucose, Chemostat, 448 generations	Kvitek and Sherlock 2013
**C646F**	S288C (1N)	E2, Bulk	0.08% Glucose, Chemostat, 448 generations	Kvitek and Sherlock 2013
**E817K**	S288C (1N)	E2, Bulk	0.08% Glucose, Chemostat, 448 generations	Kvitek and Sherlock 2013
**$^{\phi}$S509Stop /RGT1**	S288C (4N)	324	2% Raffinose, serial passage, 250 generations	Selmecki et al. Nature 2015

Supplementary Table S4. *RGT1* mutations recovered from yeast populations evolved in low glucose medium. We performed a literature search to find other reported mutations in *RGT1* that resulted from experimental evolution in carbon-limited conditions. Mutations are diagrammed in Figure 4B. The *S509Stop/RGT1* mutation (indicated by ϕ) occurred in a strain that was originally 4N but has a base ploidy of 2N after 250 generations. The nonsense mutation occurs in 1 of 2 alleles of *RGT1*.

Supplementary References

Boles E, Hollenberg CP. 1997. The molecular genetics of hexose transport in yeasts. FEMS Microbiol. Rev. 21:85–111.

Flick KM, Spielewoy N, Kalashnikova TI, Guaderrama M, Zhu Q, Chang H-C, Wittenberg C. 2003. Grr1-dependent Inactiavtion of MTH1 Mediates Glucose-induced Dissociation of Rgt1 from HTX Gene Promoters. Mol. Biol. Cell 14:3230–3241.

Kim J, Polish J, Johnston M. 2003. Specificity and Regulation of DNA Binding by the Yeast Glucose Transporter Gene Respressor Rgt1. Mol. Cell. Biol. 23:5208–5216.

Koschwanez JH, Foster KR, Murray AW. 2013. Improved use of a public good selects for the evolution of undifferentiated multicellularity. Elife 2013:1–27.

Krogh a, Larsson B, von Heijne G, Sonnhammer E. 2001. Predicting transmembrane protein topology with a hidden Markov model: application to complete genomes. J. Mol. Biol. 305:567–580.

Kvitek DJ, Sherlock G. 2013. Whole genome, whole population sequencing reveals that loss of signaling networks is the major adaptive strategy in a constant environment. PLoS Genet. 9:e1003972.

Lafuente MJ, Gancedo C, Jauniaux JC, Gancedo JM. 2000. Mth1 receives the signal given by the glucose sensors Snf3 and Rgt2 in Saccharomyces cerevisiae. Mol. Microbiol. 35:161–172.

Lagunas R. 1993. Sugar transport in Saccharomyces cerevisiae. FEMS Microbiol. Lett. 104:229–242.

Liang H, Gaber RF. 1996. A novel signal transduction pathway in Saccharomyces cerevisiae defined by Snf3-regulated expression of HXT6. Mol. Biol. Cell 7:1953–1966.

Lin Z, Li WH. 2011. Expansion of hexose transporter genes was associated with the evolution of aerobic fermentation in yeasts. Mol. Biol. Evol. 28:131–142.

Naumov G, Turakainen H, Naumova E, Aho S, Korhola M. 1990. A new family of polymorphic genes in Saccharomyces cerevisiae: α-galactosidase genes MEL1-MEL7. Mol. Gen. Genet. 224:119–128.

Ozcan S, Dover J, Rosenwald AG, Wölfl S, Johnston M. 1996. Two glucose transporters in Saccharomyces cerevisiae are glucose sensors that generate a signal for induction of gene expression. PNAS 93:12428–32.

Pasula S, Chakraborty S, Choi JH, Kim J-H. 2010. Role of casein kinase 1 in the glucose sensor-mediated signaling pathway in yeast. BMC Cell Biol. 11:17.

Polish J a., Kim J-H, Johnston M. 2005. How the Rgt1 Transcription Factor of Saccharomyces cerevisiae Is Regulated by Glucose. Genetics 169:583–594.

Raser JM, Shea EKO. 2006. Control of Stochasticity in Eukaryotic Gene Expression Jonathan. Science 304:1811–1814.

Roy A, Shin YJ, Cho KH, Kim J-H. 2013. Mth1 regulates the interaction between the Rgt1 repressor and the Ssn6-Tup1 corepressor complex by modulating PKA-dependent phosphorylation of Rgt1. Mol. Biol. Cell 24:1493–503.

Sabina J, Johnston M. 2009. Asymmetric signal transduction through paralogs that comprise a genetic switch for sugar sensing in Saccharomyces cerevisiae. J. Biol. Chem. 284:29635–43.

Selmecki AM, Maruvka YE, Richmond P a, Guillet M, Shoresh N, Sorenson AL, De S, Kishony R, Michor F, Dowell R, et al. 2015. Polyploidy can drive rapid adaptation in yeast. Nature 519:349–352.

Wenger JW, Piotrowski J, Nagarajan S, Chiotti K, Sherlock G, Rosenzweig F. 2011. Hunger artists: yeast adapted to carbon limitation show trade-offs under carbon sufficiency. PLoS Genet. 7:e1002202.
